# Recent Molecular Mechanisms and Beneficial Effects of Phytochemicals and Plant-Based Whole Foods in Reducing LDL-C and Preventing Cardiovascular Disease

**DOI:** 10.3390/antiox10050784

**Published:** 2021-05-15

**Authors:** Salman Ul Islam, Muhammad Bilal Ahmed, Haseeb Ahsan, Young-Sup Lee

**Affiliations:** 1 School of Life Sciences, BK21 FOUR KNU Creative BioResearch Group, Kyungpook National University, Daegu 41566, Korea; salman2013@knu.ac.kr (S.U.I.); muhammad786@knu.ac.kr (M.B.A.); haseeb2020@knu.ac.kr (H.A.); 2 Department of Pharmacy, Faculty of Life and Environmental Sciences, University of Peshawar, Peshawar 25120, Pakistan

**Keywords:** plant-based foods, LDL, CVD, lipid oxidation, dietary fiber, cholesterol, hyperlipidemia

## Abstract

Abnormal lipid metabolism leads to the development of hyperlipidemia, a common cause of multiple chronic disorders, including cardiovascular disease (CVD), obesity, diabetes, and cerebrovascular disease. Low-density lipoprotein cholesterol (LDL-C) currently remains the primary target for treatment of hyperlipidemia. Despite the advancement of treatment and prevention of hyperlipidemia, medications used to manage hyperlipidemia are limited to allopathic drugs, which present certain limitations and adverse effects. Increasing evidence indicates that utilization of phytochemicals and plant-based whole foods is an alternative and promising strategy to prevent hyperlipidemia and CVD. The current review focuses on phytochemicals and their pharmacological mode of actions for the regulation of LDL-C and prevention of CVD. The important molecular mechanisms illustrated in detail in this review include elevation of reverse cholesterol transport, inhibition of intestinal cholesterol absorption, acceleration of cholesterol excretion in the liver, and reduction of cholesterol synthesis. Moreover, the beneficial effects of plant-based whole foods, such as fresh fruits, vegetables, dried nuts, flax seeds, whole grains, peas, beans, vegan diets, and dietary fibers in LDL-C reduction and cardiovascular health are summarized. This review concludes that phytochemicals and plant-based whole foods can reduce LDL-C levels and lower the risk for CVD.

## 1. Introduction

**Figure 1:**
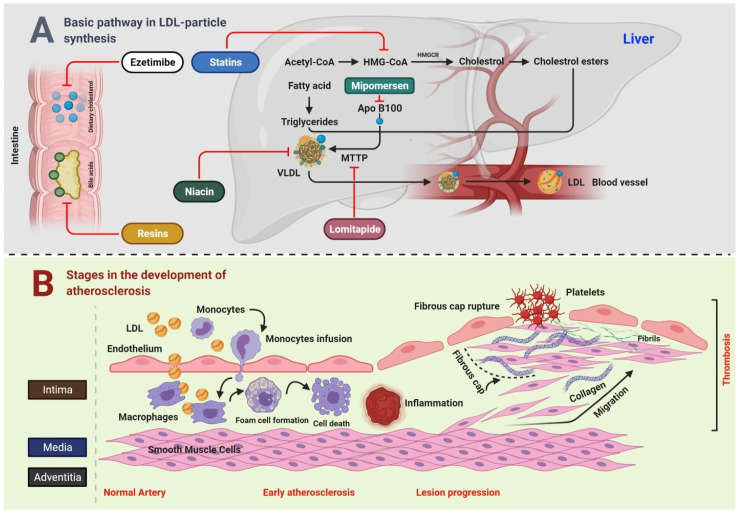
Connection between LDL and CVD.

Cholesterol is circulated in the human body by five major types of lipoproteins: high-density lipoprotein (HDL), low-density lipoprotein (LDL), intermediate-density lipoprotein (IDL), very low-density lipoprotein (VLDL), and chylomicrons [1]. The metabolism and plasma levels of cholesterol are mostly regulated by the liver. During the first step of LDL formation, intrahepatic cholesterol, either via gut absorption or de novo synthesis, is repackaged by the liver along with phospholipids, triglycerides (TGs), and proteins into VLDL particles, which then enter the general blood circulation and are converted into more cholesterol-enriched species, first IDL and then LDL, by lipoprotein lipase and cholesteryl ester transfer protein (Figure 1A). The concentrations of these circulating lipoprotein species are then regulated by the liver primarily by clearance through LDL receptors on the hepatocyte surface [2,3].

Figure 1A Bile acids and dietary cholesterol are absorbed from the lower and upper small intestine, respectively. Cholesterol absorption inhibitor, for example, ezetimibe, and bile acid sequestrants (resins) disrupt these pathways, subsequently reducing the intrahepatic cholesterol pool. The synthesis of cholesterol occurs in the liver through a multistep process that starts with acetyl-CoA. HMGCR is the rate-limiting enzyme, whose action is blocked by statins. TG are produced by esterification of fatty acids on a 3-carbon glycerol backbone. TG and esterified cholesterol are assembled by microsomal triglyceride transfer protein MTTP into nascent VLDL particles with ApoB100 on their surface, whereas MTTP and ApoB100 remain the targets for lomitapide and mipomersen, respectively. In the blood, endovascular lipases process VLDL particles to LDL particles, which are catabolized by the LDLR mainly on liver cells. Niacin inhibits the transport of VLDL. Figure 1B Key steps in the development of atherosclerosis include early atherosclerosis, lesion progression, and thrombosis. LDL play a crucial role in the development of atherosclerosis. During early atherosclerosis, monocytes are capture by the endothelial cells of the inner layer of arterial wall. The endothelial permeability assists the LDL particles in migrating into the arterial wall. Monocytes become mature, and are transformed to macrophages, which uptake the LDL particles yielding to fat-laden foam cells. Early atherosclerosis is followed by lesion progression where the smooth muscle cells move from the middle layer of the arterial wall into the tunica intima. The last step is the thrombosis which is characterized by the rapturing of the fibrous cap of a plaque and establishment of contact of blood coagulation components with the thrombogenic plaque.

## 2. Correlation of LDL Cholesterol with CVD

Several investigations provide strong evidence that LDL cholesterol (LDL-C) is a potent cardiovascular risk factor [4]. Early studies such the “Multiple Risk Factor Intervention Trial” measured total cholesterol instead of LDL-C, indicating a strong correlation between cholesterol and cardiovascular mortality [5]. However, this relationship can be assigned to LDL-C because LDL contains a major part of total cholesterol. Multiple investigations have confirmed LDL-C to be the most atherogenic lipoprotein. Studies have shown that circulating LDL particles penetrate the endothelium of arterial walls and are oxidized. Then, these oxidized LDL particles induce inflammation of the overlying endothelium and surrounding smooth muscle cells [6] (Figure 1B). Persistent elevations in circulating LDL-C levels have been directly linked to the progression from early-stage fatty streaks to advanced-stage, lipid-rich plaques. For instance, LDL receptor-deficient mice, which fail to clear LDL from the blood, have excessive LDL-C, which promoted the development of severe atherosclerosis [7], whereas mice with virtually no LDL-C did not develop atherosclerosis irrespective of diet and other risk factors for coronary heart disease (CHD) [8].

An epidemiological study demonstrated LDL-C as an independent predictor of CVD risk, as LDL-C levels > 160 mg/dL are associated with > 1.5-fold greater risk of CHD than levels < 130 mg/dL [9]. However, besides the role of LDL-C as a risk marker, researchers have also established it as a true risk factor based on investigations where inhibition of LDL-C via β-hydroxy-β-methylglutaryl coenzyme A (HMG-CoA) reductase inhibitors decreased cardiovascular events [10]. These findings have been verified by multiple large randomized controlled trials of LDL lowering like the MRC/BHF Heart Protection Study in 20,536 UK adults [11]. Most of the time, these trials focused to investigate the actions of statins, and were further supported by large meta-analyses. For instance, the Prospective Pravastatin Pooling Project (PPP) pooled the data from the West of Scotland Coronary Prevention Study (WOSCOPS), the Cholesterol and Recurrent Events trial (CARE), and the Long-term Intervention with Pravastatin in Ischemic Disease study (LIPID), providing over 100,000 person-years of follow-up [12]. Likewise, the prospective meta-analysis of the Cholesterol Treatment Trialists’ (CTT) Collaboration pooled the data from 14 randomized statin trials, containing over 90,000 individuals [13]. These trials offer exceptional statistical power for proving the potency and safety of statin therapy for a multitude of patient subgroups and endpoints.

It has been reported that statin treatment reduced the five-year incidence of major coronary events, stroke, and coronary revascularization by about one-fifth per mmol/L reduction in LDL-C [14]. Another meta-analysis from the CTT Collaboration analyzed the efficacy and safety of more intensive versus standard LDL-C lowering by statin therapy. The data were collected from 170,000 participants in a total of 26 randomized trials, which demonstrated that further decrease in LDL-C (0.51 mmol/L at one year vs. standard therapy) reduced the incidence of major coronary events by 15% [15]. Based on this information, guidelines have been established suggesting different target levels of LDL-C for different subgroups of patients. Almost all cardiovascular guidelines point to the evidence for LDL-C being both a prime cause of CHD, and a primary target of therapy [16]. Moreover, although many single-nucleotide polymorphisms (SNPs) of genes associated with increased LDL-C levels, including LDL receptor (LDLR), apolipoprotein E (ApoE), proprotein convertase subtilisin/kexin type 9 (PCSK9), and apolipoprotein B (ApoB), have been correlated with an increased risk of CVD, specific SNPs of these same genes have been associated with decreased LDL-C levels and lower risks of CVD [17,18,19,20].

At present, hyperlipidemia is primarily treated with allopathic antihyperlipidemic drugs. However, due to intolerance and adverse effects associated with these medicines, plant-based foods are important alternatives [21,22]. Plant-based foods contain various bioactive phytochemicals that can decrease LDL levels through multiple hyperlipidemia-related biological pathways. Consumption of plant-based foods has emerged as a promising and potentially cost-effective approach to decrease LDL levels while also adhering to the concept of “green” healthcare [23,24]. The following sections describe the underlying mechanisms of phytochemicals to reduce the cholesterol levels and prevent CVD.

## 3. Major Cholesterol Regulatory Mechanisms of Phytochemicals

### 3.1. Acceleration of Reverse Cholesterol Transport

Reverse cholesterol transport (RCT) is a crucial pathway that removes excess cholesterol from peripheral tissues and delivers them to the liver [25,26]. The RCT comprises of three main processes: cholesterol efflux, where excess cholesterol is removed from cells; modulation of lipoprotein, where HDL gains structural and functional changes; hepatic lipid uptake, where HDL delivers cholesterol to the liver, which is finally excreted into bile and feces [27]. In vivo investigations have demonstrated that promotion of RCT might decrease CVD and atherosclerotic plaque burden [28].

#### 3.1.1. Cholesterol Efflux

Cholesterol efflux is referred to the removal of excess cholesterol from macrophages. Studies have demonstrated the ATP-binding cassette transporter A1 (ABCA1) and ATP-binding cassette transporter G1 (ABCG1) to be the most important transporters contributing to regulate cholesterol efflux from cells. ABCA1 is responsible for the efflux to lipid-free apolipoprotein A-I (ApoA-I), whereas ABCG1 regulates efflux to mature HDL [29,30,31]. It has been reported that promotion of cholesterol efflux effectively inhibited the formation of foam cells and subsequent atherosclerosis caused by dyslipidemia [32,33].

Multiple investigations have suggested that phytochemicals such resveratrol [34], puerarin [35], leonurine [36], luteolin [37], andrographolide [38], leoligin [39], chrysin [40], and allicin [41] could enhance cholesterol efflux to HDL through ABCA1 or ABCG1. A Chawla et al. [42] reported that the PPARγ-LXR-ABCA1 pathway contributed to cholesterol efflux in macrophages. It was demonstrated that most of the above-mentioned phytochemicals increased the expressions of ABCA1 or ABCG1 through PPARγ or LXR. Moreover, previous studies have reported that quercetin-induced ABCA1 levels and cholesterol efflux were mediated by activation of TAK1-MKK3/6-p38 signaling cascade [43,44,45].

#### 3.1.2. Modulation of Lipoprotein

Besides cholesterol efflux, inhibiting lipid uptake in macrophages is another mechanism to inhibit foam cell formation, which eventually leads to suppress atherosclerotic plaque formation. CD36 (cluster of differentiation 36) and scavenger receptor class A (SR-A) are mainly responsible for uptake of lipoprotein-derived cholesterol by macrophages [46]. Several mechanisms have been described for phytochemicals through which they induce intracellular cholesterol efflux. For instance, a study reported that icariin, an active flavonol diglycoside, downregulated the CD36 expressions level through p38MAPK signaling pathway [47]. Additionally, paeonol was shown to repress the CD36 at both mRNA and protein levels by inhibiting the nuclear translocation of C—Jun [48]. Puerarin blocked the TLR4/NFκB signaling and decreased the expressions of CD36 [49]. Likewise, rographolide [38], and salvianolic acid B [50] were reported to inhibit CD36. An investigation reported that ginsenoside-Rd blocked the activity of SR-A, which caused reduction of oxidized LDL uptake and cholesterol aggregation in macrophages [51].

After removal from cells, free cholesterol is converted to cholesteryl esters by lecithin: cholesterol acyltransferase (LCAT) to form mature HDL [52]. Relevant investigations have been conducted on phytochemical is this area. Researchers have demonstrated that curcumin [53] and naringin [54] increased the RCT via LCAT and exerted anti-atherosclerosis effects.

It has been reported that cholesterol ester transporter (CETP) transfers cholesterol esters (CEs) from HDL towards ApoB-containing lipoproteins, resulting in reduced concentration of HDL and ApoA-I, while elevating the concentration of CE in VLDL and remnants [55]. As CETP elevates the concentration of VLDL and LDL-C, its specific knockdown can reduce atherosclerotic CVD [56]. It has been reported that anthocyanins could effectively inhibit the activity of CETP in humans [57].

#### 3.1.3. Hepatic Lipid Uptake

As already mentioned, that cholesterol metabolism is mostly regulated by the liver, where it takes up LDL and HDL-CE particles by LDLR and scavenger receptor class B type I (SR-BI), respectively. The LDLR binds to LDL on the cell surface. PCSK9 has been shown to post-transcriptionally downregulate the LDLR by binding to the receptor’s epidermal growth factor repeat A (EGF-A) on the cell surface and shuttling it to the lysosomes for degradation [58].

Multiple phytochemicals have been shown to alleviate atherogenesis by modulating the activity of LDLR and PCSK9. For instance, berberine was shown to upregulate the hepatic expression of LDLR and sterol regulatory element-binding protein 2 (SREBP-2), whereas it downregulated the expression of hepatocyte nuclear factor 1 [59,60]. Another investigation reported that berberine exerted anti-lipid effects by regulating hepatic LDLR and PCSK9 via the ERK signaling pathway [61]. Piseth Nhoek et al. [62] reported that flavonoid compounds, 3,7,2’-trihydroxy-5-methoxy-flavanone and skullcapflavone II, isolated from the roots of *Scutellaria baicalensis*, downregulated the PCSK9 at mRNA level via sterol regulatory element-binding protein-1 (SREBP-1). Furthermore, other phytochemicals such as curcumin [63], and tanshinone IIA [64], also modulated the activity of LDLR via downregulation of PCSK9.

**Figure 2:**
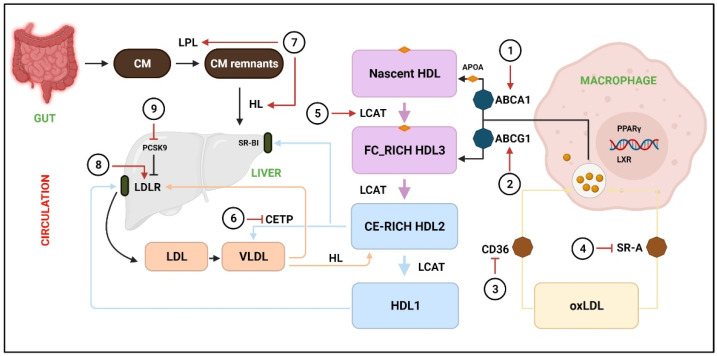
Acceleration of RCT by various phytochemicals.

Lipoprotein lipase (LPL) is the rate-limiting enzyme in the circulation of cholesterol metabolism, hydrolyzing the TG core of circulating TG-rich lipoproteins, VLDL, and chylomicrons [65]. In addition, hepatic lipase is required for the hydrolysis of triglyceride-rich lipoproteins [66]. A study reported that paeoniflorin regulated the GALNT2-ANGPTL3-LPL signaling pathway to diminish dyslipidemia in mice [67]. Yan Zhang and coworkers reported that osthole (an active constituent obtained from the fruit of *Cnidium monnieri* (L) Cusson) decreased the TC and TG in rat serum, and this effect was related with the elevated activities of LPL and hepatic lipase [68] (Figure 2).

RCT is responsible to facilitate the transport of excess cholesterol from peripheral tissues to the liver, where it is redistributed to other tissues or eliminated from the body through the gallbladder. The transport of cholesterol into macrophages occurs through CD36 and SR-A, whereas extra cholesterol is effluxed through ABCA1 and ABCG1 to the nascent HDL and free FC-rich HDL3, respectively. LCAT converts nascent HDL, FC-rich HDL3, and CE-rich HDL2 to HDL1, whereas CETP catalyzes HDL2 to VLDL. Next, HDL2 and VLDL are taken up by SR-B1 and LDLR, respectively. (1, 2) The activity of ABCA1 and ABCG1 is promoted by resveratrol [34], puerarin [35], leonurine [36], luteolin [37], andrographolide [38], leoligin [39], and chrysin [40]. (3) Downregulation of CD36 by icariin [47], paeonol [48], and puerarin [49], salvianolic acid B [50], and rographolide [38]. (4) Inhibition of SR-A by ginsenoside-Rd [51]. (5) LCAT levels are elevated by naringin [54] and curcumin [53]. (6) CETP is downregulated by crocin anthocyanins [57]. (7) LPL and HL are stimulated by paeoniflorin [67] and osthole [68]. (8) LDLR expression is increased by berberine [59,60]. (9) PCSK9 expression are downregulated by 3,7,2’-trihydroxy-5-methoxy-flavanone, skullcapflavone II [62], curcumin [63], and tanshinone IIA [64]. CM = chylomicron; CM remnants = chylomicron remnants; HL = hepatic lipase; FC = free cholesterol; CE = cholesterol ester; oxLDL = Oxidized low-density lipoprotein; LXR = Liver X receptor.

### 3.2. Inhibition of Intestinal Cholesterol Absorption

Absorption of cholesterol refers to the transfer of intraluminal cholesterol into enterocytes or thoracic duct lymph [69]. The food substances and bile enter from the intestinal lumen into enterocytes via the transmembrane protein Niemann-Pick C1 like1 (NPC1L1) [70]. Inside the enterocytes, free cholesterol is esterified to CEs by an enzyme called acyl CoA: cholesterol acyltransferase-2 (ACAT)-2 in the endoplasmic reticulum [71]. Afterward, CEs and TG, under the action of MTTP, form chylomicrons, which are then secreted into the lymphatic system [72]. Studies have reported that inhibition of intestinal cholesterol absorption effectively lowered the plasma LDL-C level [73] and reduced the risk of CVD [74]. Thus, it is necessary to block excessive absorption of cholesterol from the diet and bile [75].

#### 3.2.1. Cholesterol Uptake Inhibition

Phytochemicals show blood lipid-lowering effects and inhibit cholesterol uptake mainly by targeting NPC1L1. It has been shown that downregulation of NPC1L1 considerably reduces intestinal cholesterol absorption [70], which is modulated by SREBP-2 [76] and LXR [77]. Curcumin has been shown to block cholesterol uptake by binding to the NPC1L1-related transporter [78,79]. A study reported the curcumin response elements to be present in the region between −291 and +56 of NPC1L1 promoter [80]. Additionally, curcumin was shown to inhibit NPC1L1 pathways by activating the SREBP2 transcription factor [80]. Jun Zou and Dan Feng reported that lycopene, the predominant carotenoid in tomatoes, blocked intestinal cholesterol absorption by blockade of the LXRα pathway [81]. Another study reported that ankaflavin and monascin suppressed the protein levels of NPC1L1 associated with small intestine tissue lipid absorption [82].

#### 3.2.2. Enhancement of Cholesterol Esterification

**Figure 3:**
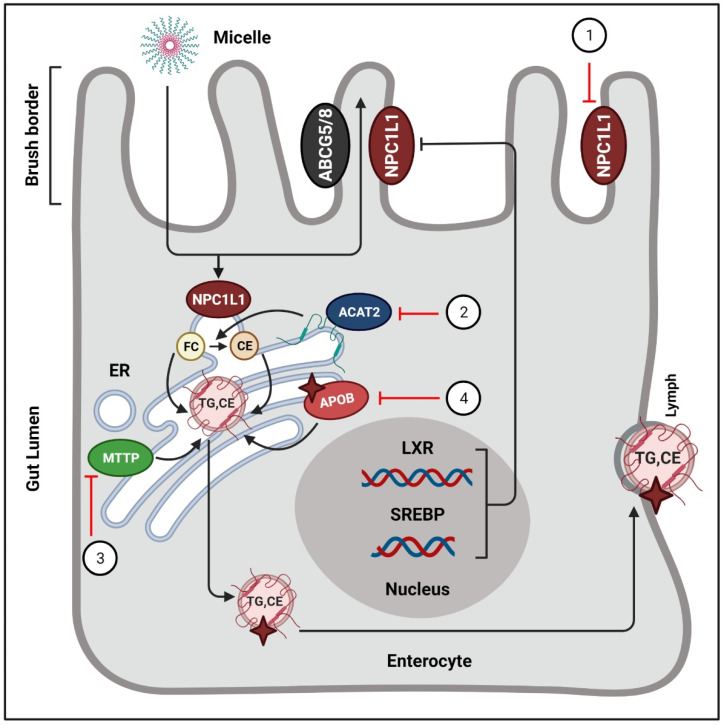
Phytochemicals block the absorption of intestinal cholesterol.

Studies have reported that ACAT2 exhibits a strong connection with the plasma cholesterol levels and catalyzes the formation of cholesteryl ester in enterocytes [71,83]. Phytochemicals such as oleanolic acid and ursolic acid have been reported to reduce cholesterol levels by inhibiting the activity of ACAT [84,85]. Additionally, downregulation of MTTP is associated with decreased ApoB secretion and chylomicron assemblage. Flavonoids, such as hesperetin [86], quercetin [87], taxifolin [88], tangeretin [89], and naringenin [86] have shown MTTP inhibitory activities. Ioanna Vallianou and Margarita Hadzopoulou-Cladaras reported that camphene, in response to a decrease in the intracellular cholesterol exerts, upregulated the expression of SREBP-1 and blocked the activity of MTTP [90]. It was shown that Tanshinone IIA repressed the MTTP’s transcripts and stimulated cellular ApoB proteasomal degradation [91]. Likewise, nobiletin [92], tangeretin [92], and lignin [93] have been shown to reduce the secretion of ApoB. Collectively, phytochemicals inhibit excessive cholesterol absorption and reduce blood lipid levels by downregulating NPC1L1, MTTP, ACAT2, ApoB, LXR, and SREBP (Figure 3).

In intestinal lumen, cholesterol incorporates into bile salt micelles and diffuses to the brush border membrane of enterocytes via NPC1L1. Inside the intestinal cells, free cholesterols are esterified by ACAT-2, and afterwards they enter the lymphatic system in the form of chylomicrons. Phytochemicals exert their actions to regulate these processes. (1) NPC1L1 is suppressed by curcumin [78,79], lycopene [81], monascin, and ankaflavin [82]. (B) ACAT2 is targeted by oleanolic acid, and ursolic acid [84,85]. (C) The activity of MTTP is blocked by hesperetin [86], quercetin [87], taxifolin [88], tangeretin [89], and naringenin [86]. (4) Tanshinone IIA stimulates the proteasomal degradation of cellular ApoB [91]. ER = endoplasmic reticulum; FC = free cholesterol; CE = cholesterol ester; ABCG5/8 = ATP-binding cassette sub-family G member 5/8; TG = triglycerides

### 3.3. Promotion of Cholesterol Excretion in the Liver

Hepatic cholesterol, after conversion to bile acids, is removed from the body through biliary secretion [94]. Cholesterol 7 alpha-hydroxylase (CYP7A1) is the first and rate-limiting enzyme in the bile acid synthesis pathway, which performs a crucial role in maintaining cholesterol homeostasis [95,96]. The activity of CYP7A1 has been shown to be promoted by phytochemicals such as catechins and gypenosides [97,98]. Furthermore, utilization and excretion of cholesterol was improved by palmatine (main alkaloids in *Coptis chinensis*) and jatrorrhizine (extracted from *Rhizoma coptidis*) through upregulation of CYP7A1 mRNA [99,100]. Another phytochemical called columbamine, obtained from *Rhizoma coptidis*, indirectly transactivated CYP7A1 by the stimulation of hepatocyte nuclear factor 4-alpha and fetoprotein transcription factor, resulting in enhanced cholesterol catabolism and bile acids secretion [101]. A study demonstrated that punicalagin and ellagic acid extracted from pomegranate enhanced cholesterol metabolism in human hepatocytes by activating the PPARγ-CYP7A1 signaling [102] (Figure 2).

### 3.4. Inhibition of Cholesterol Synthesis

The synthesis of cholesterol is regulated through an elegant system of feedback inhibition that senses intracellular cholesterol and eventually regulates several proteins participating in cholesterol homeostasis [103,104]. Squalene synthase (SQS) and HMGCR remain the crucial enzymes involved in cholesterol homeostasis, and the genes of these enzymes are regulated by SREBP-2 [105]. Likewise, AMP-activated protein kinase (AMPK) remains a key sensor in the regulation of lipid metabolism [106]. It was reported that alteration of AMPK restricted the rate of HMG-CoA expression, which, in turn, regulated the synthesis of cholesterol [107].

**Figure 4:**
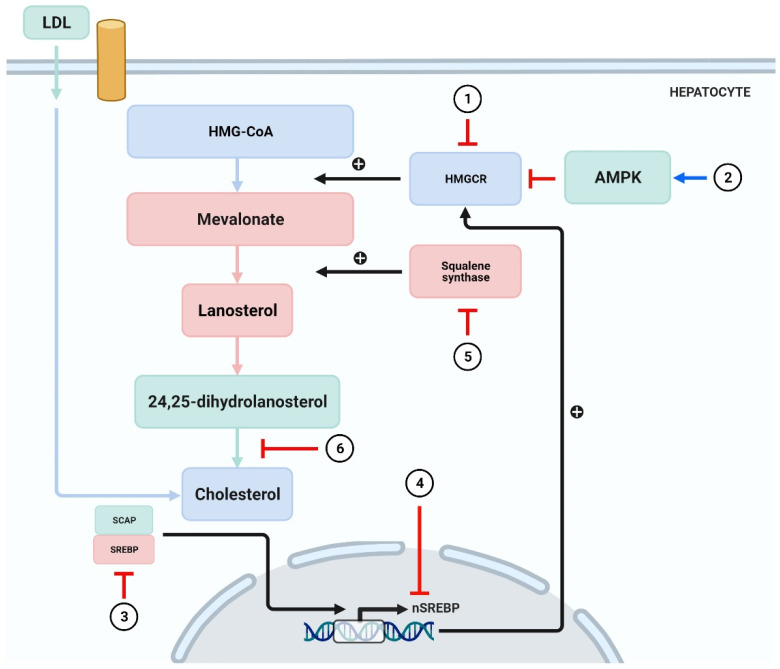
Phytochemicals inhibit the synthesis of cholesterol.

Studies have described several phytochemicals such as curcumin [108], leoligin [109], ODP-Ia [110], puerarin [111], and geraniol [112], which could suppress the synthesis of cholesterol through inhibition of HMGCR. Moreover, certain phytochemicals have been shown to inhibit synthesis of cholesterol by activating the AMPK [62,63,64,65,66,67,68,69,70]. SREBPs contribute to the intake of cholesterol and exert regulatory effect on genes encoding HMGCR [113]. Emodin has been reported to inhibit the transcription of SREBP-2 and subsequently suppress the biosynthesis of cholesterol [114]. T Grand-Perret et al. [115] reported that activation of SCAP/SREBP signaling pathway remarkably inhibited cholesterol biosynthesis. Another study reported that (−)-epicatechin and tetramethylpyrazine blocked the SCAP/SREBP-1c pathway, which resulted in the amelioration of atherosclerosis and lipid metabolism disorders [116,117]. Hassan Hajjaj and coworkers [118] reported that 26-oxygenosterol, obtained from *Ganoderma lucidum*, downregulated lanosterol 14 alpha-demethylase, which is responsible for the conversion of 24, 25-dihydrolanosterol into cholesterol. A study demonstrated SQS to be an attractive target for antihyperlipidemic drugs due to its contribution in cholesterol synthesis [119]. Yankun Chen et al. [120] reported that cynarin inhibited SQS and led to decrease the TG levels (Figure 4).

(**1**) HMGCR is blocked by curcumin [108], leoligin [109], ODP-Ia [110], puerarin [111], and geraniol [112]. (**2**) Activation of AMPK by phytochemicals such as curcumin, tanshinone IIA, and paeoniflorin leads to inhibition of cholesterol synthesis [62,63,64,65,66,67,68,69,70]. (**3**,**4**) Emodin inhibits the transcription of SREBP-2 [114], whereas (−)-epicatechin and tetramethylpyrazine block the SCAP/SREBP-1c pathway [116,117]. (**5**) 26-oxygenosterol promotes the activity of SQS and subsequently block cholesterol synthesis [118]. (**6**) Cynarin exerts inhibitory action in the process where 24,25-sdiydrolanosterol forms cholesterol [120]. SCAP = sterol regulatory element binding protein (SREBP) cleavage-activating protein.

## 4. Plant-Based Whole Foods Reducing LDL-C and Contributing to Prevent CVD

Low intake of fruits and vegetables was reportedly responsible for ~25.5 million premature deaths globally in 2013 [121]. Vegetables and fruits are good sources of various beneficial substances, such as dietary fiber, minerals, vitamins, and antioxidant entities, which can collectively reduce the risk of chronic disorders and total mortality and exert beneficial effects on the gut microbiota [122,123,124]. The intake of vegetables, fruits, and dietary fiber has been reported to have positive effects on serum cholesterol levels and platelet aggregation [125]. The results of a meta-analysis found that a daily increase of 200 g of fruits and vegetables reduced the relative risks of CHD, stroke, and CVD by 8–16%, 13–18%, and 8–13%, respectively [126]. Moreover, daily consumption of ~500 g of fruits and vegetables has been correlated with a 22% lower risk of CVD than with a dietary intake of 0–40 g/day [126].

### 4.1. Grapes (Vitis Vinifera)

Grapes are rich sources of phenolic compounds, with an appreciable anthocyanin content of ~46% [127]. Although anthocyanins convey antioxidant, anti-inflammatory, antihypertensive, and antiplatelet activities [128], some studies have reported conflicting results regarding the effects on lipid profiles [129,130].

The effects of grape juice on dyslipidemia were studied using a mouse model homozygous for the absence of the LDLR gene (LDLR^−/−^) and fed a hyperlipidemic diet. During this study, 30 male mice (12 weeks old) were assigned to one of three groups (10 mice/group): the HL group, which received a high-fat diet; the HLU group, fed a high-fat diet and grape juice (2 g/kg/day), and the HLS group, which received a high-fat diet along with simvastatin (20 mg/kg/day). Blood pressure, lipid levels, glycemic and insulinemic profiles, and C-reactive protein levels were determined. It was noted that the 60-day outcomes of the HLU and HLS group were similar, as the addition of grape juice diminished dyslipidemia and effectively elevated HDL-C levels. Moreover, left ventricular hypertrophy and arterial hypertension was prevented in the HLU group. These results suggest that dietary grape juice can potentially prevent CVD [131].

Various clinical trials have found that grape polyphenols are effective against cholesterolemia. For instance, van Mierlo et al. [132] reported that, compared with a placebo, the intake of grape polyphenols (800 mg/day) for 2 weeks led to decreased TC and TG levels. Similar outcomes were obtained by another investigation on 60 healthy volunteers who received 700 mg/day of a polyphenol-rich grape extract supplement for one month [133]. A study of 44 pre-or postmenopausal women found that dietary supplementation of lyophilized grape powder (39 g/day for 4 weeks) effectively reduced serum levels of LDL-C, ApoE, ApoB, and TGs [134]. Furthermore, administration of grape polyphenols for three weeks and consumption of red wine for one month was reported to reduce LDL-C levels and the risk of CVD [135,136].

### 4.2. Cranberries (Vaccinium Macrocarpon)

Cranberries are a rich source of flavonoids (flavanols, flavan3-ols, and anthocyanins) and phenolic acids (ellagic, benzoic, and hydroxycinnamic acids), which contribute to reducing the risk of CVD through antioxidant, anti-inflammatory, and antithrombotic mechanisms [137]. Wilson et al. first reported the LDL-protective properties of cranberry juice (pressed berries), as 0.10% cranberry juice suppressed the formation of thiobarbituric acid reactive substances via Cu^2+^-induced oxidation of LDL [138]. Another study reported that dietary intake of 2.8 mg/g of cold-pressed cranberry seed oil inhibited LDL oxidation [139]. Besides improving the resistance of LDL to oxidation, cranberry extract has also been shown to increase cholesterol uptake by HepG2 cells and to enhance the synthesis of LDL receptors, which resulted in accelerated cholesterol excretion in vivo [140]. In another study of the effects of cranberry juice powder on blood cholesterol levels, pigs with familial hypercholesterolemic (FH) were fed a diet supplemented with 47 g/day of citric acid and 57 g/day of fructose for two weeks. On day 15, 150 g/day of cranberry juice powder was added and continued for four weeks. Total blood cholesterol, HDL, and LDL levels were observed weekly. At baseline, LDL levels in the FH pigs were 11-fold greater than in normal pigs (428 vs. 37 mg/dL, respectively), whereas total blood cholesterol was sevenfold greater (458 vs. 67 mg/dL, respectively). At the end of the investigation, the LDL levels decreased to 94 mg/dL and total blood cholesterol to 92 mg/dL in the FH pigs. These findings indicate that cranberry juice powder can decrease cholesterol levels in hypercholesterolemic individuals [141].

### 4.3. Pomegranate (Punica Granatum)

Pomegranates contain several potent antioxidants (anthocyanins and tannins), which act as effective anti-atherogenic agents [142]. Daily consumption of pomegranate juice has been shown to reduce serum LDL-C and TG levels and to increase HDL-C levels [143]. The considerable amounts of steroidal compounds in pomegranate seed oil were reported to decrease cholesterol levels [144]. A study of the effect of concentrated pomegranate juice on cholesterol profiles of type-2 diabetes patients with hyperlipidemia reported that daily intake of 40 g of pomegranate for 8 weeks effectively reduced TC and LDL-C levels, as well as the TC/HDL-C and LDL-C/HDL-C ratios [145]. Moreover, pomegranate juice was found to reduce LDL accumulation and increase HDL levels by 20% in humans, whereas a 90% decrease in LDL levels was noted in mice [146]. Al-Moraie et al. [147] reported that consumption of 1–5 mL/kg of pomegranate juice for 28 days effectively decreased LDL-C, VLDL-C, TC, and TG levels while elevating the expression of antioxidant enzymes and HDL-C levels. A study investigating the correlation of punicalagin (the main polyphenol in pomegranate) with ApoB100 that surrounds LDL particles showed that punicalagin bound to ApoB100 at low concentrations (0.25–4 μM) and stimulated LDL influx to macrophages (up to 2.5-fold) in a dose-dependent manner. The study further demonstrated that LDL influx to macrophages occurred specifically via the LDL receptor. The most important fact demonstrated by this investigation was that the interaction of punicalagin with LDL led specifically to LDL influx to the macrophages without their conversion into foam cells. The study concluded that upon binding to ApoB100, punicalagin induced LDL influx to macrophages, thereby decreasing circulating cholesterol levels [148].

### 4.4. Apple (Malus Domestica)

The effect of apple polyphenols on blood lipid profile has been the focus of numerous studies. A study, while investigating the cholesterol-lowering effect of five different apple species (annurca apple, red delicious, Granny Smith, fuji, and golden delicious) in mildly hypercholesterolaemic healthy subjects, showed that annurca apples exerted the most significant effects; allowing a reduction in TC and LDL-C levels by 8.3% and 14.5%, respectively, while increased HDL-C level by 15.2% [149]. Another study observed the effects of eating two apples/day for four months on blood cholesterol levels. The results showed that blood cholesterol was lowered by 14.5% as compared with the untreated group, whereas HDL-C levels were increased by 15% [150]. However, other than a change in oxidized LDL levels, there was no reduction in blood cholesterol [151]. Another study of lyophilized apples found that administration of 0.21–1.43 g of polyphenols daily for one month did not improve the cardiovascular health of obese patients [152]. Similar results were obtained from another investigation where 300 g golden delicious apple per day for eight weeks increased the serum levels of VLDL and TG, but had no effect on TC, LDL-C, HDL-C, LDL/HDL ratio, and ApoB [153]. Hence, dietary supplementation of apple as whole fruit led to inconsistent results. Although the polyphenolic content of apples was found to lower blood cholesterol levels and LDL oxidation, these data are insufficient to conclude that apples, as a dietary nutraceutical, can lower plasma cholesterol [154,155].

### 4.5. Dried Nuts

Nuts are rich in polyunsaturated fatty acids, phytosterols, polyphenolics, and fiber [156]. Many studies have reported that daily consumption of nuts can improve cardiovascular health [157,158,159]. Almonds, pistachios, and walnuts are the most highly consumed nuts worldwide [160]. In a previous meta-analysis, of all edible nuts, pistachios were found to improve blood lipid profiles and effectively reduce serum TG, TC, and lipoprotein levels. Walnuts were second to pistachios in lowering TGs and cholesterol. Controlled levels of LDL, TGs, and TC are important serum markers of cardiovascular health [60,128,161]. Almonds were found to reduce LDL levels more effectively compared with other markers. Mechanisms responsible for the lipid-lowering abilities of nuts include reduced absorption of dietary cholesterol and increased bile production. Nuts are also reported to have an inhibitory effect against HMG-CoA, which is required for biosynthesis of cholesterol via acetyl CoA. Multiple bioactive constituents (phytosterols and fiber) may also individually convey cardiovascular benefits [162,163,164]. Ellagitannins and lutein in nuts have also been shown to reduce blood lipid levels [165,166]. At the cellular level, nuts have been reported to influence the expression levels of several miRNAs associated with lipid metabolism and uptake [167,168].

Several meta-analyses of the cardiovascular benefits of nuts have reported similar results. However, there have been conflicting reports, as one study claimed that walnuts and pistachios do not lower serum TG levels [169]. As a possible explanation for these conflicting findings, some studies evaluated nut-enriched foods containing other ingredients, such as skimmed milk and components of Mediterranean diets, which could influence the effects of nuts [169,170,171,172]. Nonetheless, the superiority of pistachios in lowering blood cholesterol is reportedly due to the greater content of β-carotene, γ-tocopherol and lutein [156].

The reported effects of nut-enriched diets on body weight varies among studies, as some reported a slight reduction in adipose tissue content in response to daily intake of nut-enriched foods [173], whereas others found that the high fat and calories of nuts can cause weight gain [160]. In a meta-analysis of 34 studies, only one reported significant weight loss, whereas the others found no significant correlation between a nut-enriched diet and body weight [174,175].

When compared with lipid-lowering drugs, such as statins, the effect of nuts on cholesterol seems to be quite modest. Moreover, the duration of most of the studies were relatively short (<six months), whereas dyslipidemia disorders are chronic. Hence, well-designed studies of larger populations for greater durations are required to accurately evaluate the effects of nut-enriched diets on cardiovascular health. Another concern is the reliance on self-reporting the frequency and quantity of nut-enriched diets, which may be misleading. Furthermore, many studies failed to report daily intake of nuts on regular basis. In addition, nuts, such as pecans, peanuts, and Brazil nuts, were often not included in the studies. Most meta-analyses did not conduct sensitivity analysis due to limited data. Hence, based on the available published studies with sensitivity analysis, diets enriched with pistachios and walnuts have more favorable lipid-lowering effects than other nut-enriched diets. However, the quality of evidence is debatable due to the several shortcomings addressed earlier [176].

### 4.6. Fruits of Opuntia Spp.

Many studies have reported that consumption of various fruits of *Opuntia* spp. can significantly lower TC [177,178]. A recent study also reported the lipid-lowering effects of juice consumption at 150 mL/day for two weeks consecutively in both healthy and hyperlipidemic populations [179]. The processes thought to be involved in this effect was a reduction in fat absorption in the intestine, increased bile synthesis and secretion, and increased density of LDL receptors at cholesterol uptake sites. The high fiber content of fruits has been suggested to be the primary lipid-lowering ingredient. Reduced enterohepatic recirculation of bile is another factor responsible for indirect reduction in blood cholesterol levels [180]. Other studies have suggested that pectin derived from fruit also promotes the production of bile by increasing the biosynthesis of chenodeoxycholic acid and increased uptake of LDL from the blood. Other studies have reported such effects without reporting the quantity of daily fiber intake. Hence, due to the phytochemical constituents and fiber content, *Opuntia* spp. are good candidates for managing CVD [177].

### 4.7. Flax Seeds

Flax seeds are rich in dietary fiber, which consists of pentose- and hexose-based hydrophilic polymers, such as arabinoxylans, galactose, ketose (fructose), pectin, and omega-3 fatty acids, which form high consistency solutions in the gut [181]. Beverages containing flax fibers have been reported to reduce fasting levels of TC and LDL by 12% and 15%, respectively [182]. Another study reported that consumption of roasted flax seed powder for three months significantly reduced serum levels of TG, TC, VLDL, and LDL [183].

Lignans isolated from the flax seeds are being extensively studied for their hypocholesterolemic effects. A study was conducted for two months, in patients with high blood cholesterol, to observe the effects of administering the dietary secoisolariciresinol diglucoside (SDG) from flax seeds on lipid profile. The results exhibited that dose of 600 mg SDG was sufficient to reduce TC and LDL up to 24%. Authors concluded that flax seed lignans had significant anti hypercholestrolemic effects [184]. Another study was conducted which involved ingestion of whole flax and sunflower seeds by a special population of hypercholestrolemic postmenopausal women. Patients were given 38 g of one diet for six weeks followed by switching of diet for another six weeks. The washout period between two dietary regimens was a two-week interval. Flax seed diet was able to reduce TC and LDL up to 6.9% and 14.7% respectively. Marked reduction in the concentrations of lipoprotein A (7.4%) was also observed. Reduction in TC and LDL due to sunflower seeds was lower than flax seeds. The authors were of the view that the above effects of whole seeds of both plants were due to the presence of linoleic acids, and fibers present in adequate amounts [185]. Another study investigated the effects of ground flax seeds on the lipid profile of patients taking statins to control blood cholesterol. The randomised double blind study conducted for 12 months involved the administration of 30g ground seeds to 58 patients. A significant reduction in LDL (15%) was observed after one month of diet therapy. 11% reduction in TC was observed after 6 mon of flax seeds intake. The authors also reported fading of flax seeds effects on cholesterol lowering after 6 months of treatment. Combining flax seeds diet with statins caused a consistent reduction in LDL by 8.5% after 12 months [186].

### 4.8. Whole Grains

Whole grains, such as rice, corn, barley, and rye, are rich sources of fiber. An analysis of 64 studies [183] concluded that regular intake of oat reduced serum TG and LDL levels by up to 19% and 23%, respectively. Among these 64 studies, some also described the favorable HDL-elevating effects of oat. Another review of 24 studies reported that intake of whole grain foods also lowered serum TC and LDL levels, with the most dominant effect on TC [183].

Yet another study conducted on 12754 individuals to observe their dietary habits with respect to adequate intake of whole grains (more than 3 oz per day) and their daily dose of statins. The researchers reported that one fourth of total individuals were regular in consuming whole grains and statins for the total duration of study (12 months). They also reported that concomitant use of dietary and pharmacological means of cholesterol control was better than either of the two regimens used alone. Therefore, inclusion of whole grains in adequate amounts lessens the odds of patients in developing more severe forms of cardiovascular emergencies [187]. Just as two or more servings of refined sugars and carbohydrates have been associated with greater risk of developing CVDs, daily servings of whole grains (two or more) have been associated with 10–20% lower risk of developing CVDs [188]. Another study assessed the effect of whole grains by analysing the data from 24 studies. Whole grain intake caused reduction in triglycerides and cholesterol levels without affecting HDL [189]. A study assessed the effects of a diet containing whole grains, fruits and vegetables on CVD risk and weight gain. A total of 75 overweight women were randomized and given one of the above-mentioned diets for 10 weeks. The results, when examined after 10 weeks, showed that women who took whole grains for 10 weeks displayed better lipid control profile (lowered LDL), lower hypertension, and greater weight reduction than those who were given fruits and vegetables [190]. Another study tried to analyse the effect of specific grains against whole grains in reducing CVD risk. A prospective study conducted on 2329 old age (late 50s) individuals with previous history of myocardial infarction (MI). Examination of eating habits of these individuals followed by risk estimation using Cox proportional hazard model revealed that whole grains are effective in lowering the risk of future CVDs. Among individual grains, rye and oats were found to be specifically more effective than other individual grains in lowering risk of MI and other CVDs [191].

### 4.9. Soy Components

Soy proteins are one of the plant based food components approved by FDA for their health claim regarding their cholesterol reducing effects. Researchers studied 46 trials after the approval to judge if the health claim still holds value. Of 46, 41 had data on LDL-C while 43 had data on TC. Analysis showed that 25 mg daily dose of soy proteins for 6 weeks caused reduction in LDL-C and TC by 4.76 and 6.41 mg/dL respectively. This reduction amounted to 3–4% reduced LDL-C by consumption of soy proteins, in adults [192]. Combining phospholipids, soy fibers and proteins revealed a synergistic effect on lowering blood cholesterol than soy proteins alone [193]. Another study conducted in population of postmenopausal women, reported that soy phytosterols (4g) taken in combination with fibers and soy proteins induce greater antihyperlipidemic effects than soy proteins alone [194].

Soy isoflavones have been reported to regulate lipogenesis, beta oxidation of fatty acids, and lipolysis by inhibiting the Akt/mTORC1 pathway [99]. In addition, isoflavones were reported to reduce serum TC and LDL levels by 1.7% and 3.6%, respectively, in hypercholesterolemic patients with a lesser effect in normal individuals [195]. Another study found greater lipid-lowering effects in earlier periods of treatment with overall reductions in serum TC, LDL, and TG levels of 3.7%, 5.25%, and 7.27%, respectively. Moreover, soy isoflavones were reported to increase the amount of beneficial HDL by 3.03% [196]. A meta-analysis confirmed that whole soya from four weeks to one year reduced more serum LDL compared with processed soya-extracts [197].

Consumption of 2 g/day of soybean leaf extract for 12 weeks was reported to reduce serum TG levels in overweight subjects with mildly high blood glucose levels [110], whereas another study showed that 70 mg/day for three months decreased serum TG levels in postmenopausal women [198].

Soy isoflavones include daidzein, genistein, and glycitein, which occur as glycosides. The linkage is broken by digestive enzymes to liberate the active aglycone portion of glycosides. A study of the lipid-lowering effects of daidzein (soy isoflavone) at 40 and 80 mg/day for six months found that the effects on TG levels were dose-dependent [199]. In the colon, daidzein is converted to S-equol. In a previous study, 10 mg/day of S-equol was found to reduce blood LDL levels and the cardio-ankle vascular index [200]. Although daidzein is metabolized in the colon, 30–50% of the Caucasian population had dissimilar results among the different groups. Likewise, genistein at 54 mg/day for 12 months increased HDL levels from 46.4 mg/dl to 56.8 mg/dl (22.4% increase) and decreased LDL levels from 108.8 mg/dl to 78.7 mg/dl (27.66% decrease) in postmenopausal women [201].

### 4.10. Vegetarian Diet

Vegetarian diets excludes meat, poultry and fish. Vegan diet, in addition to the exclusions of vegetarian diet, also excludes eggs and dairy products. Health benefits of vegetarian diets are due to the presence of adequate quantities of *n*−6 fatty acids, fiber, carotenoids, folate, ascorbic acid, vitamin E and magnesium. On the other hand, these diets lack sufficient protein, vitamin A, B12 and zinc. Vegan foods are low in calcium too due to absence of dairy products [202]. A scientific opinion suggested that a person does not need to become vegan or vegetarian to improve their cardiovascular health but slight changes in dietary components and habits might do the needful [203].

Reducing consumption of meat is thought to be correlated with improved cardiovascular health [204]. Vegetarian and Mediterranean-type diets have been recommended as health imparting diets by the American College of Cardiology/American Heart Association. The diets of Africans are largely plant-based, which have been associated with a lower prevalence of CVD [205]. A study conducted in the US reported a clear correlation between meat consumption and CVD [206]. Another significant study showed that a vegetarian diet reduces the risk of CVD by 32%, as vegetables have anti anti-inflammatory and antioxidant effects due to the prevalence of carotenoids, flavonoids, and other polyphenols [207]. A main drawback of such diets is the lower availability of proteins and certain minerals, especially zinc and calcium. Some researchers have argued that the presence of trypsin inhibitors (pulses, tomato), lyso-alanine (in processed vegetables), and glucosinolates in plant-based foods can also cause harm to the body due to the addition of non-vegetarian diets; thus, a slight reduction in consumption of vegetarian diets is recommended [208,209,210].

A meta-analytic study of 11 trials was conducted to observe the effects of vegetarian diets on lipid profile. Random effect model was used for determination of net changes in lipid profile. Vegetarian diets caused significant reduction in TC and LDL-C without having significant effect on lowering triglycerides [211]. A meta analytic and prospective study reported that vegetarian diets continued for 5 years or more reduced the risk of mortality from CHD by 24% [212]. A study conducted in Taiwanese patients to compare the effects of vegetarian diet against an omnivorous diet on CVDs reported that vegetarian diet was more effective in lowering TC and LDL-C. No effect was observed on HDL-C and TGs. However, homocysteine levels (indicative of folate and B12 deficiency) were higher in the vegetarian population [213].

## 5. Dietary Fiber

**Figure 5:**
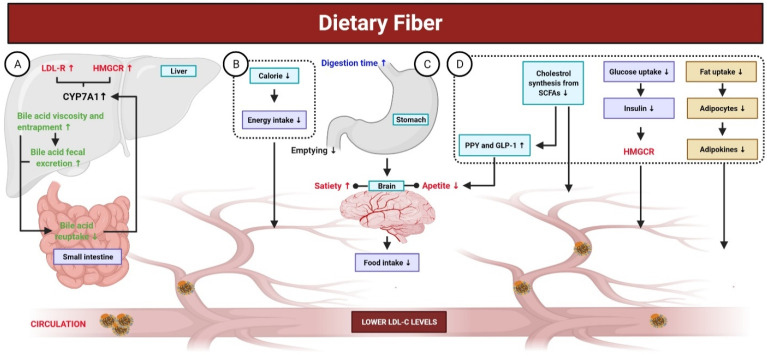
Mechanisms of dietary fiber in the gut. Dietary fibers are carbohydrate polysaccharides; not hydrolyzed by digestive enzymes in gastrointestinal tract and are associated with a low risk for CVD. (**A**) Dietary fibers elevate the fecal excretion of bile acid, decrease its re-uptake in the small intestine, and inhibit bile acid permeation. A reduced enterohepatic pool of bile acid stimulates the CYP7A (the rate-limiting enzyme involved in the production of bile acid), which, in turn, enhances liver uptake of LDL-C from blood through the upregulation of LDL-R and HMGCR. (**B,C**) Dietary fibers exhibit fewer calories and their consumption results in a prolonged digestion time with delayed gastric emptying. Additionally, it leads to an increase in bulk-forming and satiety, as well as viscosity-induced reduced absorption of cholesterol, which eventually lower the concentration of LDL-C. (**D**) Reduced cholesterol synthesis from SCFAs, produced by dietary fibers fermentation in the intestine, contributes to decrease the concentration of LDL-C. Certain SCFAs (propionate) enhances the release of PYY and GLP-1, both of which contribute to reduce LDL-C concentration. Additionally, dietary fibers intake leads to reduced fat uptake, altering the production of adipokines (TNF-α, resistin, and leptin), which play key roles in lipid metabolism and improving cholesterol concentration. The viscosity of dietary fibers reduces the intestinal absorption of glucose, leading to decreased secretion of insulin. Insulin is responsible for stimulating HMGCR; thus, lower insulin could decrease the concentration of LDL-C.

Fibers are carbohydrate polysaccharides that are not hydrolyzed by digestive enzymes in gastrointestinal tract. Low fiber intake has been associated with a higher risk of CVD. In contrast, fiber-rich foods, such as fruits and vegetables, have been associated with a low risk for CVD [214]. This correlation is attributed to the increased bile secretion induced by fibers, lower fatty acid biosynthesis, increased insulin sensitivity at target tissues, feeling satiated due to increased indigestible bulk in the intestine, lower cravings, and improved bowel habits [215,216]. Beta glucan has been shown to increase the consistency of stomach and intestinal contents and reduce enterohepatic recirculation of bile via adsorption of bile acids by viscose fiber dispersion and inhibition of micelle formation for lipid emulsification, which leads to indirect reduction in blood cholesterol levels (Figure 5). Moreover, pectin and guar gum have been reported to possess such effects [217]. A previous meta-analysis of 15 studies with a large combined cohort of ~1 million reported a correlation between mortality and fiber consumption and found that daily intake of ~30 g/day of dietary fiber reduced the risk of mortality due to CVDs by 23% [218]. The source of fiber also affects its physiological function [219], as soluble fibers have cholesterol-lowering effects, whereas insoluble fibers can prevent sudden increases in blood glucose levels [220]. Beta glucan is a soluble fiber found in oat and barley. Many studies have associated the intake of 3 g beta glucan per day with lower serum TG, cholesterol, and LDL levels (5–10%) [186]. In a murine model of hypertension, dietary fiber was found to play a role in the downregulation of early growth response 1, which is a transcription factor that regulates many genes and pathways involved in the progression of CVD [219,221]. Another meta-analysis reported that intake of 3 g/day of beta glucan contained in fibrous foods decreased LDL levels with no effect on HDL and TGs [222]. Another study of 2295 individuals over a period of ~5 years found that fibrous foods, such as pulses, fruits, and vegetables, reduced the risk of CVD [223].

### 5.1. Epidemiological Evidence on Dietary Fiber and Health

Risk factors for CVD include obesity, diabetes, and hypertension. A prospective cohort study reported that consumption of 7 g/day of total fiber reduced the risk of CVD by 9% [224]. The same amount has been reported to decrease the risk of type-2 diabetes up to 6%, especially with whole grain foods [225]. Additionally, the study also revealed that three servings of whole grain bread reduced the risk of diabetes by 32% [225]. The ability of fibers (beta glucan) to reduce hypertension has also been observed in healthy individuals, although the reduction has been modest, as systolic pressure was reduced by 2·9 mmol Hg and diastolic pressure by 1·5 mm Hg [226]. Another systematic review deduced from various trials that the fibers content in a cereal bowl of porridge with an oat snack reduced blood cholesterol; 0·15 mmol in unclassified participants and 0·20 mmol in participants with hypercholesterolaemia [227].

Butyrate is used as an energy source by various bacteria for survival. Other SCFAs also affect fatty acid biosynthesis and oxidation. Once absorbed into the portal circulation, propionate and acetate can reduce hepatic synthesis of cholesterol and lipids [228]. Moreover, the receptor-mediated activation of SCFAs causes change in the activity of protein kinase A, which affects lipid metabolism. Propionate has also been reported to cause the release of peptide YY and glucagon-like peptide 1, which affects the metabolism and muscle uptake of free fatty acids and reduces the severity of hepatic steatosis. Adipokines are chemical messengers responsible for the metabolism of body fat and adipocytes. Fiber consumption has been reported to reduce the production of adipokines, which may result in lower production of fatty tissue. These chemical messengers are also involved in glycemic metabolic control [229,230].

### 5.2. Fiber Supplements

It has been estimated that risk of CHD in a population reduces by 2% for every 1% decrease in cholesterol [231]. Estimates indicate that in the two decades (1980–2000), health conscious behavior led to significant reductions in cholesterol intake that caused 33% reduction in mortality due to CHD in US [3,232]. Intake of dietary fibers in adequate amount is found in 5% adults. Therefore, supplements are required to provide concentrated form of fiber. Among many marketed products, psyllium, β-glucan and inulin are famous as natural supplements. Semisynthetic processed fibers include wheat dextrin, cyclodextrin and methylcellulose [233]. β-glucan containing foods can reduce the cholesterol levels of consumers. Viscous fibers such as those containing β-glucan have greater tendency to reduce cholesterol absorption. A study was conducted on 345 individuals to assess the effect of processed β-glucan supplement on LDL-C. Results depicted that administration of 3 g high viscosity β-glucan (achieved via low heat and pressurized processing) caused 5.5% reduction in LDL-C against placebo (wheat fiber) [234]. Cyclodextrin, which is produced from corn, is reported to reduce TC by 5%, LDL by 6.7%, and ApoB by 5.6%. This reduction in lipids occurs due to the presence of cyclodextrin in the diet without changing any other dietary component, although this effect was more pronounced in patients with high serum TG and cholesterol levels [235]. A study of the effect of 2 g/day of cyclodextrin on post prandial TG levels found that the dose was sufficient to reduce blood TG levels after a fatty meal [236].

## 6. Concluding Remarks and Future Directions

Scientists believe that CVD can be prevented through adoption of healthy lifestyle habits and optimal improvements in risk factors including high levels of LDL-C. Phytochemicals and plant-based whole foods can achieve better LDL-C control, and their addition is highly recommended to preventive strategies either for all subjects or more focused to patients with CVD. Our review focused the beneficial effects of phytochemicals and plant-based whole foods in the management of hyperlipidemia and CVD prevention.

Phytochemicals regulate the process of lipid metabolism and multi-target intervention, which occur in cholesterol biosynthesis, absorption, transport, and elimination. NPC1L1, HMGCR, PCSK9, and CYP7A1 remain the key molecules involved in these processes. In addition, during these processes, transcription factors such as LXR, SREBP, PPARα, and PPARγ actively participate and mediate the lipid-lowering actions of phytochemicals. The outcomes regarding the utilization of phytochemicals are promising and emphasize the potential indication of these phytochemicals in different categories of patients. The phytochemicals described in this review are diverse, including polyphenols (pomegranate), alkaloids (berberine), flavonoids (taxifolin, quercetin), and saponins (ginsenoside). These phytochemicals are mostly safe and well tolerated. Collectively, the phytochemicals can reduce the levels of LDL-C and prevent CVD by regulating different metabolic pathways.

Plant-based whole foods are known for their LDL-C lowering potential and cardiovascular health benefits [237,238]. These foods include vegetables, fruits, legumes, nuts, whole grains, dietary fibers, and seeds [157,237]. Plant-based whole foods are abundant in dietary fibers, unsaturated fatty acids, plant proteins, multiple micronutrients like vitamins, and phytochemicals like polyphenols and phytosterols [237]. These foods influence the development of CVD either directly or indirectly by different underlying mechanisms. For example, replacing saturated fats with unsaturated fatty acids in the diet is known to reduce LDL-C [239,240]. It has been reported in multiple observational studies and randomized controlled trials, that replacing saturated fatty acids with vegetable oil polyunsaturated fatty acids decreases the risk of CVD [239].

Dietary fibers, especially viscous soluble fibers like beta-glucan, inhibit the intestinal absorption of cholesterol and re-absorption of bile acids, producing SCFAs in the colon, which may influence hepatic cholesterol synthesis, and eventually result in LDL-C-lowering effect [241]. A significant correlation has been established between dietary fibers intake and lower risk of all-cause mortality [218] and mortality from CVD as well as CHD [218,241]. Phytosterols have also been shown to block the intestinal cholesterol absorption and reduce the concentrations of circulating LDL-C [242]. Siying S Li et al. [98] showed that replacing animal protein with plant protein resulted in lower LDL-C.

Of note, all plant-based whole foods are not necessarily effective in lowering CVD risk because not all plant-based whole foods exhibit beneficial CV effects [237,243]. As we mentioned earlier that golden delicious apple per day for eight weeks elevated the serum levels of VLDL and TG, and did not show considerable effect on TC, LDL-C, HDL-C, LDL/HDL ratio, and ApoB [153]. Similarly, foods pattern with more refined grains have been linked with a higher CVD risk [244]. Therefore, the quality of plant-based whole foods and food components are of prime importance.

Although plant-based foods are generally perceived positively because of their health benefits, there are also multiple barriers that hinder the switch to, and maintenance of a plant-based diet. Common hurdles encompass health concerns that plant-based foods may lack specific nutrients, enjoyment of eating meat and animal-based foods, and reluctance to change dietary behavior [245,246]. Similarly, there are certain other limitations and unresolved issues. For example, several bioactive components of plant-based foods have not yet been characterized; thus, further intensive investigations are needed. Moreover, the dosage of plant-based foods greatly varies, which subsequently leads to inconsistent outcomes. It is worth mentioning that plant-based foods can reduce LDL-C when utilized in defined effective nontoxic quantities. Hence, further studies are needed to optimize the doses of processed products, extracts, and compounds obtained from plant-based foods to enhance efficacy and minimize toxicity and adverse effects. Moreover, to obtain the maximum advantages of plant-based foods for the management of LDL-C and CVD, current knowledge about bioavailability and toxicity in human must be improved. The antihyperlipidemic effects of plant-based foods need to be investigated at the cellular and molecular levels in high-quality studies with systemic and in-depth analyses. Furthermore, large-scale clinical trials are also required to assure the hypolipidemic effects. Despite these shortcoming, gaps, and limitations that have to be addressed, plant-based foods are an effective strategy to manage LDL-C and CVD.

## Abbreviations

Acetyl CoA	Acetyl coenzyme A
Akt	Protein kinase B
ApoB	Apolipoprotein B
ApoB100	Apolipoprotein B100
ApoE	Apolipoprotein E
CHD	Coronary heart disease
CVD	Cardiovascular disease
CYP7A1	Cytochrome P450 Family 7 Subfamily A Member 1
ERK	Extracellular Signal-Regulated Kinase
GLP-1	Glucagon-like peptide-1
HDL	High-density lipoprotein
HDL-C	HDL cholesterol
HMG-CoA	β-hydroxy-β-methylglutaryl coenzyme A
HMGCR	β-hydroxy-β-methylglutaryl coenzyme A reductase
IDL	Intermediate-density lipoprotein
LDL	Low-density lipoprotein
LDL-C	LDL Cholesterol
LDLR	Low-density lipoprotein receptor
LDLR^-/-^	Deleted low-density lipoprotein receptor
LXR	Liver X receptor
MKK3	Mitogen-activated protein kinase kinase 3
mTORC1	Mammalian target of rapamycin complex 1 or mechanistic target of rapamycin complex 1
MTTP	Microsomal triglyceride transfer protein large subunit
PPARα	Peroxisome proliferator-activated receptor alpha
PPARβ	Peroxisome proliferator-activated receptor beta
PPARγ	Peroxisome proliferator-activated receptor gamma
PPY	Peptide YY
ROS	Reactive oxygen species
SCFAs	Short-chain fatty acids
S-equol	7-Hydroxy-3-(4′-hydroxyphenyl)-chroman
SNP	Single-nucleotide polymorphism
SOD	Superoxide dismutase
SREBP-1c	Sterol regulatory element-binding protein-1c
TAK1	Mitogen-activated protein kinase kinase kinase 7
TC	Total cholesterol
TGTC	TriglyceridesTotal cholesterol
VLDL TG	Very low-density lipoprotein Triglycerides
VLDL	Very low-density lipoprotein

## References

[1] Saeed A., Feofanova E.V., Yu B., Sun W., Virani S.S., Nambi V., Coresh J., Guild C.S., Boerwinkle E., Ballantyne C.M. (2018). Remnant-Like Particle Cholesterol, Low-Density Lipoprotein Triglycerides, and Incident Cardiovascular Disease. J. Am. Coll. Cardiol..

[2] Ramasamy I. (2014). Recent advances in physiological lipoprotein metabolism. Clin. Chem. Lab. Med..

[3] Wadhera R.K., Steen D.L., Khan I., Giugliano R.P., Foody J.M. (2016). A review of low-density lipoprotein cholesterol, treatment strategies, and its impact on cardiovascular disease morbidity and mortality. J. Clin. Lipidol..

[4] Yu D., Liao J.K. (2021). Emerging views of statin pleiotropy and cholesterol lowering. Cardiovasc. Res..

[5] Stamler J., Wentworth D., Neaton J.D. (1986). Is relationship between serum cholesterol and risk of premature death from coronary heart disease continuous and graded? Findings in 356,222 primary screenees of the Multiple Risk Factor Intervention Trial (MRFIT). JAMA.

[6] Borén J., Chapman M.J., Krauss R.M., Packard C.J., Bentzon J.F., Binder C.J., Daemen M.J., Demer L.L., Hegele R.A., Nicholls S.J. (2020). Low-density lipoproteins cause atherosclerotic cardiovascular disease: Pathophysiological, genetic, and therapeutic insights: A consensus statement from the European Atherosclerosis Society Consensus Panel. Eur. Heart J..

[7] Véniant M.M., Sullivan M.A., Kim S.K., Ambroziak P., Chu A., Wilson M.D., Hellerstein M.K., Rudel L.L., Walzem R.L., Young S.G. (2000). Defining the atherogenicity of large and small lipoproteins containing apolipoprotein B100. J. Clin. Investig..

[8] Lieu H.D., Withycombe S.K., Walker Q., Rong J.X., Walzem R.L., Wong J.S., Hamilton R.L., Fisher E.A., Young S.G. (2003). Eliminating Atherogenesis in Mice by Switching Off Hepatic Lipoprotein Secretion. Circulation.

[9] Wilson P.W.F., D’Agostino R.B., Levy D., Belanger A.M., Silbershatz H., Kannel W.B. (1998). Prediction of Coronary Heart Disease Using Risk Factor Categories. Circulation.

[10] Iqbal D., Khan M.S., Khan M.S., Ahmad S., Hussain M.S., Ali M. (2015). Bioactivity guided fractionation and hypolipidemic property of a novel HMG-CoA reductase inhibitor from Ficus virens Ait. Lipids Health Dis..

[11] Group HPSC (2002). MRC/BHF Heart Protection Study of antioxidant vitamin supplementation in 20 536 high-risk individuals: A randomised placebo-controlled trial. Lancet.

[12] Sacks F.M., Tonkin A.M., Shepherd J., Braunwald E., Cobbe S., Hawkins C.M., Keech A., Packard C., Simes J., Byington R. (2000). Effect of pravastatin on coronary disease events in subgroups defined by coronary risk factors: The Prospective Pravastatin Pooling Project. Circulation.

[13] Unit ES (2005). Efficacy and safety of cholesterol-lowering treatment: Prospective meta-analysis of data from 90 056 participants in 14 randomised trials of statins. Lancet.

[14] Poess J., Boehm M., Laufs U. (2009). Are the guidelines correct? Should all patients with coronary heart disease or diabetes be treated with a statin?. Med. Klin. Munich Ger. 1983.

[15] Baigent C., Blackwell L., Emberson J., Holland L.E., Reith C., Bhala N., Peto R., Barnes E.H., Keech A., Simes J. (2010). Efficacy and Safety of More Intensive Lowering of LDL Cholesterol: A Meta-Analysis of Data from 170,000 Participants in 26 Randomised Trials.

[16] (2002). National Cholesterol Education Program (U.S.) Expert Panel on Detection, Evaluation, and Treatment of High Blood Cholesterol in Adults (Adult Treatment Panel III): "Third Report of the National Cholesterol Education Program (NCEP) Expert Panel on Detection, Evaluation, and Treatment of High Blood Cholesterol in Adults (Adults Treatment Panel III) Final report". Circulation.

[17] Willer C.J., Sanna S., Jackson A.U., Scuteri A., Bonnycastle L.L., Clarke R., Heath S.C., Timpson N.J., Najjar S.S., Stringham H.M. (2008). Newly identified loci that influence lipid concentrations and risk of coronary artery disease. Nat. Genet..

[18] Kathiresan S., Melander O., Anevski D., Guiducci C., Burtt N.P., Roos C., Hirschhorn J.N., Berglund G., Hedblad B., Groop L. (2008). Polymorphisms Associated with Cholesterol and Risk of Cardiovascular Events. N. Engl. J. Med..

[19] Investigators MIGC (2014). Inactivating mutations in NPC1L1 and protection from coronary heart disease. N. Engl. J. Med..

[20] Blood I., Crosby J., Peloso G.M., Auer P.L., Crosslin D.R., Stitziel N.O., Lange L.A., Lu Y. (2014). TG, HDL Working Group of the Exome Sequencing Project NH, Lung, Institute B. Loss-of-function mutations in APOC3, triglycerides, and coronary disease. N. Engl. J. Med..

[21] Kumar A., Mosa K.A., Ji L., Kage U., Dhokane D., Karre S., Madalageri D., Pathania N. (2018). Metabolomics-assisted biotechnological interventions for developing plant-based functional foods and nutraceuticals. Crit. Rev. Food Sci. Nutr..

[22] Mahamuni S.P., Khose R.D., Menaa F., Badole S.L. (2012). Therapeutic approaches to drug targets in hyperlipidemia. BioMedicine.

[23] Hlaing T.T., Park A. (2013). Hyperlipidaemia. Medicine.

[24] George V.C., Dellaire G., Rupasinghe H.V. (2017). Plant flavonoids in cancer chemoprevention: Role in genome stability. J. Nutr. Biochem..

[25] Rosenson R.S., Brewer H.B., Davidson W.S., Fayad Z.A., Fuster V., Goldstein J., Hellerstein M., Jiang X.C., Phillips M.C., Rader D.J. (2012). Cholesterol efflux and atheroprotection: Advancing the concept of reverse cholesterol transport. Circulation.

[26] Rader D.J., Alexander E.T., Weibel G.L., Billheimer J., Rothblat G.H. (2009). The role of reverse cholesterol transport in animals and humans and relationship to atherosclerosis. J. Lipid Res..

[27] Favari E., Chroni A., Tietge U.J., Zanotti I., Bernini F. (2015). Cholesterol Efflux and Reverse Cholesterol Transport. High Density Lipoproteins.

[28] Feig J.E., Hewing B., Smith J.D., Hazen S.L., Fisher E.A. (2014). High-density lipoprotein and atherosclerosis regression: Evidence from preclinical and clinical studies. Circ. Res..

[29] Yvan-Charvet L., Wang N., Tall A.R. (2010). Role of HDL, ABCA1, and ABCG1 Transporters in Cholesterol Efflux and Immune Responses. Arter. Thromb. Vasc. Biol..

[30] Du X.-M., Kim M.-J., Hou L., Le Goff W., Chapman M.J., Van Eck M., Curtiss L.K., Burnett J.R., Cartland S.P., Quinn C.M. (2015). HDL Particle Size Is a Critical Determinant of ABCA1-Mediated Macrophage Cellular Cholesterol Export. Circ. Res..

[31] Yue P., Chen Z., Nassir F., Bernal-Mizrachi C., Finck B., Azhar S., Abumrad N.A. (2010). Enhanced hepatic apoA-I secretion and peripheral efflux of cholesterol and phospholipid in CD36 null mice. PLoS ONE.

[32] Mody P., Joshi P.H., Khera A., Ayers C.R., Rohatgi A. (2016). Beyond coronary calcification, family history, and C-reactive protein: Cholesterol efflux capacity and cardiovascular risk prediction. J. Am. Coll. Cardiol..

[33] Ye D., Lammers B., Zhao Y., Meurs I., Van Berkel T.J.C., Van Eck M. (2011). ATP-binding cassette transporters A1 and G1, HDL metabolism, cholesterol efflux, and inflammation: Important targets for the treatment of atherosclerosis. Curr. Drug Targets.

[34] Ye G., Chen G., Gao H., Lin Y., Liao X., Zhang H., Liu X., Chi Y., Huang Q., Zhu H. (2019). Resveratrol inhibits lipid accumulation in the intestine of atherosclerotic mice and macrophages. J. Cell. Mol. Med..

[35] Li C.H., Gong D., Chen L.Y., Zhang M., Xia X.D., Cheng H.P., Huang C., Zhao Z.W., Zheng X.L., Tang X.E. (2017). Puerarin promotes ABCA1-mediated cholesterol efflux and decreases cellular lipid accumulation in THP-1 macrophages. Eur. J. Pharmacol..

[36] Jiang T., Ren K., Chen Q., Li H., Yao R., Hu H., Lv Y.-C., Zhao G.-J. (2017). Leonurine Prevents Atherosclerosis Via Promoting the Expression of ABCA1 and ABCG1 in a Pparγ/Lxrα Signaling Pathway-Dependent Manner. Cell. Physiol. Biochem..

[37] Francisco V., Figueirinha A., Costa G., Liberal J., Ferreira I., Lopes M.C., García-Rodríguez C., Cruz M.T., Batista M.T. (2016). The Flavone Luteolin Inhibits Liver X Receptor Activation. J. Nat. Prod..

[38] Lin H.-C., Lii C.-K., Chen H.-C., Lin A.-H., Yang Y.-C., Chen H.-W. (2018). Andrographolide Inhibits Oxidized LDL-Induced Cholesterol Accumulation and Foam Cell Formation in Macrophages. Am. J. Chin. Med..

[39] Wang L., Ladurner A., Latkolik S., Schwaiger S., Linder T., Hošek J., Palme V., Schilcher N., Polanský O., Heiss E.H. (2016). Leoligin, the Major Lignan from Edelweiss (Leontopodium nivale subsp. alpinum), Promotes Cholesterol Efflux from THP-1 Macrophages. J. Nat. Prod..

[40] Wang S., Zhang X., Liu M., Luan H., Ji Y., Guo P., Wu C. (2015). Chrysin inhibits foam cell formation through promoting cholesterol efflux from RAW264.7 macrophages. Pharm. Biol..

[41] Lin X.-L., Hu H.-J., Liu Y.-B., Hu X.-M., Fan X.-J., Zou W.-W., Pan Y.-Q., Zhou W.-Q., Peng M.-W., Gu C.-H. (2017). Allicin induces the upregulation of ABCA1 expression via PPARγ/LXRα signaling in THP-1 macrophage-derived foam cells. Int. J. Mol. Med..

[42] Chawla A., Boisvert W.A., Lee C.-H., Laffitte B.A., Barak Y., Joseph S.B., Liao D., Nagy L., Edwards P.A., Curtiss L.K. (2001). A PPARγ-LXR-ABCA1 Pathway in Macrophages Is Involved in Cholesterol Efflux and Atherogenesis. Mol. Cell.

[43] Ren K., Jiang T., Zhao G.-J. (2018). Quercetin induces the selective uptake of HDL-cholesterol via promoting SR-BI expression and the activation of the PPARγ/LXRα pathway. Food Funct..

[44] Sun L., Li E., Wang F., Wang T., Qin Z., Niu S., Qiu C. (2015). Quercetin increases macrophage cholesterol efflux to inhibit foam cell formation through activating PPARγ-ABCA1 pathway. Int. J. Clin. Exp. Pathol..

[45] Chang Y.-C., Lee T.-S., Chiang A.-N. (2012). Quercetin enhances ABCA1 expression and cholesterol efflux through a p38-dependent pathway in macrophages. J. Lipid Res..

[46] Li X.-Y., Kong L.-X., Li J., He H.-X., Zhou Y.-D. (2013). Kaempferol suppresses lipid accumulation in macrophages through the downregulation of cluster of differentiation 36 and the upregulation of scavenger receptor class B type I and ATP-binding cassette transporters A1 and G1. Int. J. Mol. Med..

[47] Yang H., Yan L., Qian P., Duan H., Wu J., Li B., Wang S., Wang S. (2015). Icariin Inhibits Foam Cell Formation by Down-Regulating the Expression of CD36 and Up-Regulating the Expression of SR-BI. J. Cell. Biochem..

[48] Li X., Zhou Y., Yu C., Yang H., Zhang C., Ye Y., Xiao S. (2015). Paeonol suppresses lipid accumulation in macrophages via upregulation of the ATP-binding cassette transporter A1 and downregulation of the cluster of differentiation 36. Int. J. Oncol..

[49] Zhang H., Zhai Z., Zhou H., Li Y., Li X., Lin Y., Li W., Shi Y., Zhou M.S. (2015). Puerarin inhibits oxLDL-induced macrophage activation and foam cell formation in human THP1 macrophage. BioMed Res. Int..

[50] Bao Y., Wang L., Xu Y., Yang Y., Wang L., Si S., Cho S., Hong B. (2012). Salvianolic acid B inhibits macrophage uptake of modified low density lipoprotein (mLDL) in a scavenger receptor CD36-dependent manner. Atherosclerosis.

[51] Li J., Xie Z.-Z., Tang Y.-B., Zhou J.-G., Guan Y.-Y. (2011). Ginsenoside-Rd, a purified component from panax notoginseng saponins, prevents atherosclerosis in apoE knockout mice. Eur. J. Pharmacol..

[52] Acuña-Aravena M., Cohen D.E. (2020). Lipoprotein Metabolism and Cholesterol Balance. Liver Biol. Pathobiol..

[53] Ganjali S., Blesso C.N., Banach M., Pirro M., Majeed M., Sahebkar A. (2017). Effects of curcumin on HDL functionality. Pharmacol. Res..

[54] Rotimi S.O., Adelani I.B., Bankole G.E., Rotimi O.A. (2018). Naringin enhances reverse cholesterol transport in high fat/low streptozocin induced diabetic rats. Biomed. Pharmacother..

[55] Chapman M.J., Le Goff W., Guerin M., Kontush A. (2010). Cholesteryl ester transfer protein: At the heart of the action of lipid-modulating therapy with statins, fibrates, niacin, and cholesteryl ester transfer protein inhibitors. Eur. Heart J..

[56] Shrestha S., Wu B.J., Guiney L., Barter P.J., Rye K.-A. (2018). Cholesteryl ester transfer protein and its inhibitors. J. Lipid Res..

[57] Qin Y., Xia M., Ma J., Hao Y., Liu J., Mou H., Cao L., Ling W. (2009). Anthocyanin supplementation improves serum LDL- and HDL-cholesterol concentrations associated with the inhibition of cholesteryl ester transfer protein in dyslipidemic subjects. Am. J. Clin. Nutr..

[58] Lagace T.A. (2014). PCSK9 and LDLR degradation: Regulatory mechanisms in circulation and in cells. Curr. Opin. Lipidol..

[59] Li H., Dong B., Park S.W., Lee H.-S., Chen W., Liu J. (2009). Hepatocyte Nuclear Factor 1α Plays a Critical Role in PCSK9 Gene Transcription and Regulation by the Natural Hypocholesterolemic Compound Berberine. J. Biol. Chem..

[60] Jia Y.-J., Xu R.-X., Sun J., Tang Y., Li J.-J. (2014). Enhanced circulating PCSK9 concentration by berberine through SREBP-2 pathway in high fat diet-fed rats. J. Transl. Med..

[61] Cao S., Xu P., Yan J., Liu H., Liu L., Cheng L., Qiu F., Kang N. (2019). Berberrubine and its analog, hydroxypropyl-berberrubine, regulate LDLR and PCSK9 expression via the ERK signal pathway to exert cholesterol-lowering effects in human hepatoma HepG2 cells. J. Cell. Biochem..

[62] Nhoek P., Chae H.-S., Masagalli J.N., Mailar K., Pel P., Kim Y.-M., Choi W.J., Chin Y.-W. (2018). Discovery of Flavonoids from Scutellaria baicalensis with Inhibitory Activity against PCSK 9 Expression: Isolation, Synthesis and Their Biological Evaluation. Molecules.

[63] Tai M.-H., Chen P.-K., Chen P.-Y., Wu M.-J., Ho C.-T., Yen J.-H. (2014). Curcumin enhances cell-surface LDLR level and promotes LDL uptake through downregulation of PCSK9 gene expression in HepG2 cells. Mol. Nutr. Food Res..

[64] Chen H.-C., Chen P.-Y., Wu M.-J., Tai M.-H., Yen J.-H. (2016). Tanshinone IIA Modulates Low Density Lipoprotein Uptake via Down-Regulation of PCSK9 Gene Expression in HepG2 Cells. PLoS ONE.

[65] Wang H., Eckel R.H. (2009). Lipoprotein lipase: From gene to obesity. Am. J. Physiol. Metab..

[66] Bos G., Snijder M.B., Nijpels G., Dekker J.M., Stehouwer C.D., Bouter L.M., Heine R.J., Jansen H. (2005). Opposite Contributions of Trunk and Leg Fat Mass with Plasma Lipase Activities: The Hoorn Study. Obes. Res..

[67] Xiao H.-B., Liang L., Luo Z.-F., Sun Z.-L. (2018). Paeoniflorin regulates GALNT2-ANGPTL3-LPL pathway to attenuate dyslipidemia in mice. Eur. J. Pharmacol..

[68] Zhang Y., Xie M.L., Zhu L.J., Gu Z.L. (2007). Therapeutic effect of osthole on hyperlipidemic fatty liver in rats 3. Acta Pharmacol. Sin..

[69] Sesorova I.S., Dimov I.D., Kashin A.D., Sesorov V.V., Karelina N.R., Zdorikova M.A., Beznoussenko G.V., Mironov A.A. (2021). Cellular and sub-cellular mechanisms of lipid transport from gut to lymph. Tissue Cell.

[70] Altmann S.W., Davis H.R., Zhu L.-J., Yao X., Hoos L.M., Tetzloff G., Iyer S.P.N., Maguire M., Golovko A., Zeng M. (2004). Niemann-Pick C1 Like 1 Protein Is Critical for Intestinal Cholesterol Absorption. Science.

[71] Lee R.G., Willingham M.C., Davis M.A., Skinner K.A., Rudel L.L. (2000). Differential expression of ACAT1 and ACAT2 among cells within liver, intestine, kidney, and adrenal of nonhuman primates. J. Lipid Res..

[72] Sirwi A., Hussain M.M. (2018). Lipid transfer proteins in the assembly of apoB-containing lipoproteins. J. Lipid Res..

[73] Davidson M.H., Voogt J., Luchoomun J., Decaris J., Killion S., Boban D., Glass A., Mohammad H., Lu Y., Villegas D. (2013). Inhibition of intestinal cholesterol absorption with ezetimibe increases components of reverse cholesterol transport in humans. Atherosclerosis.

[74] Pirillo A., Catapano A.L., Norata G.D. (2016). Niemann-Pick C1-Like 1 (NPC1L1) inhibition and cardiovascular diseases. Curr. Med. Chem..

[75] Wang D.Q.-H. (2007). Regulation of Intestinal Cholesterol Absorption. Annu. Rev. Physiol..

[76] Alrefai W.A., Annaba F., Sarwar Z., Dwivedi A., Saksena S., Singla A., Dudeja P.K., Gill R.K. (2007). Modulation of human Niemann-Pick C1-like 1 gene expression by sterol: Role of sterol regulatory element binding protein 2. Am. J. Physiol.-Gastrointest. Liver Physiol..

[77] Duval C., Touche V., Tailleux A., Fruchart J.-C., Fievet C., Clavey V., Staels B., Lestavel S. (2006). Niemann–Pick C1 like 1 gene expression is down-regulated by LXR activators in the intestine. Biochem. Biophys. Res. Commun..

[78] Feng D., Ohlsson L., Duan R.-D. (2010). Curcumin inhibits cholesterol uptake in Caco-2 cells by down-regulation of NPC1L1 expression. Lipids Health Dis..

[79] Feng D., Zou J., Zhang S., Li X., Lu M. (2017). Hypocholesterolemic Activity of Curcumin Is Mediated by Down-regulating the Expression of Niemann-Pick C1-like 1 in Hamsters. J. Agric. Food Chem..

[80] Kumar P., Malhotra P., Ma K., Singla A., Hedroug O., Saksena S., Dudeja P.K., Gill R.K., Alrefai W.A. (2011). SREBP2 mediates the modulation of intestinal NPC1L1 expression by curcumin. Am. J. Physiol.-Gastrointest. Liver Physiol..

[81] Zou J., Feng D. (2015). Lycopene reduces cholesterol absorption through the downregulation of Niemann-Pick C1-like 1 in Caco-2 cells. Mol. Nutr. Food Res..

[82] Lee C.-L., Wen J.-Y., Hsu Y.-W., Pan T.-M. (2013). Monascus-Fermented Yellow Pigments Monascin and Ankaflavin Showed Antiobesity Effect via the Suppression of Differentiation and Lipogenesis in Obese Rats Fed a High-Fat Diet. J. Agric. Food Chem..

[83] Afonso M.S., Machado R.M., Lavrador M.S., Quintao E.C.R., Moore K.J., Lottenberg A.M. (2018). Molecular Pathways Underlying Cholesterol Homeostasis. Nutrients.

[84] Lin Y., Vermeer M.A., Trautwein E.A. (2011). Triterpenic Acids Present in Hawthorn Lower Plasma Cholesterol by Inhibiting Intestinal ACAT Activity in Hamsters. Evid.-Based Complement. Altern. Med..

[85] Wang Y., Yi X., Ghanam K., Zhang S., Zhao T., Zhu X. (2014). Berberine decreases cholesterol levels in rats through multiple mechanisms, including inhibition of cholesterol absorption. Metabolism.

[86] Wilcox L.J., Borradaile N.M., De Dreu L.E., Huff M.W. (2001). Secretion of hepatocyte apoB is inhibited by the flavonoids, naringenin and hesperetin, via reduced activity and expression of ACAT2 and MTP. J. Lipid Res..

[87] Casaschi A., Wang Q., Richards A., Theriault A. (2002). Intestinal apolipoprotein B secretion is inhibited by the flavonoid quercetin: Potential role of microsomal triglyceride transfer protein and diacylglycerol acyltransferase. Lipids.

[88] Casaschi A., Rubio B.K., Maiyoh G.K., Theriault A.G. (2004). Inhibitory activity of diacylglycerol acyltransferase (DGAT) and microsomal triglyceride transfer protein (MTP) by the flavonoid, taxifolin, in HepG2 cells: Potential role in the regulation of apolipoprotein B secretion. Atherosclerosis.

[89] Kurowska E.M., Manthey J.A., Casaschi A., Theriault A.G. (2004). Modulation of hepG2 cell net apolipoprotein B secretion by the citrus polymethoxyflavone, tangeretin. Lipids.

[90] Vallianou I., Hadzopoulou-Cladaras M. (2016). Camphene, a Plant Derived Monoterpene, Exerts Its Hypolipidemic Action by Affecting SREBP-1 and MTP Expression. PLoS ONE.

[91] Kang Y.-J., Jin U.-H., Chang H.-W., Son J.-K., Lee S.H., Son K.-H., Chang Y.-C., Lee Y.-C., Kim C.-H. (2008). Inhibition of microsomal triglyceride transfer protein expression and atherogenic risk factor apolipoprotein B100 secretion by tanshinone IIA in HepG2 cells. Phytotherapy Res. Int. J. Devoted Pharmacol. Toxicol. Eval. Nat. Prod. Deriv..

[92] Lin Y., Vermeer M.A., Bos W., Van Buren L., Schuurbiers E., Miret-Catalan S., Trautwein E.A. (2011). Molecular Structures of Citrus Flavonoids Determine Their Effects on Lipid Metabolism in HepG2 Cells by Primarily Suppressing ApoB Secretion. J. Agric. Food Chem..

[93] Norikura T., Mukai Y., Fujita S., Mikame K., Funaoka M., Sato S. (2010). Lignophenols Decrease Oleate-Induced Apolipoprotein-B Secretion in HepG2 Cells. Basic Clin. Pharmacol. Toxicol..

[94] Wang D.Q.H., Portincasa P., Tso P. (2017). Transintestinal cholesterol excretion: A secondary, nonbiliary pathway contributing to reverse cholesterol transport. Hepatology.

[95] Pullinger C.R., Eng C., Salen G., Shefer S., Batta A.K., Erickson S.K., Verhagen A., Rivera C.R., Mulvihill S.J., Malloy M.J. (2002). Human cholesterol 7α-hydroxylase (CYP7A1) deficiency has a hypercholesterolemic phenotype. J. Clin. Investig..

[96] Li T., Matozel M., Boehme S., Kong B., Nilsson L.-M., Guo G., Ellis E., Chiang J.Y.L. (2010). Overexpression of cholesterol 7α-hydroxylase promotes hepatic bile acid synthesis and secretion and maintains cholesterol homeostasis. Hepatology.

[97] Lee M.-S., Park J.-Y., Freake H., Kwun I.-S., Kim Y. (2008). Green tea catechin enhances cholesterol 7α-hydroxylase gene expression in HepG2 cells. Br. J. Nutr..

[98] Lu Y., Du Y., Qin L., Wu D., Wang W., Ling L., Ma F., Ling H., Yang L., Wang C. (2018). Gypenosides Altered Hepatic Bile Acids Homeostasis in Mice Treated with High Fat Diet. Evid.-Based Complement. Altern. Med..

[99] Ning N., He K., Wang Y., Zou Z., Wu H., Li X., Ye X. (2015). Hypolipidemic Effect and Mechanism of Palmatine from Coptis chinensis in Hamsters Fed High-Fat diet. Phytother. Res..

[100] Wu H., He K., Wang Y., Xue D., Ning N., Zou Z., Ye X., Li X., Wang D., Pang J. (2014). The antihypercholesterolemic effect of jatrorrhizine isolated from Rhizoma Coptidis. Phytomedicine.

[101] Wang Y., Han Y., Chai F., Xiang H., Huang T., Kou S., Han B., Gong X., Ye X. (2016). The antihypercholesterolemic effect of columbamine from Rhizoma Coptidis in HFHC-diet induced hamsters through HNF-4α/FTF-mediated CYP7A1 activation. Fitoterapia.

[102] Lv O., Wang L., Li J., Ma Q., Zhao W. (2016). Effects of pomegranate peel polyphenols on lipid accumulation and cholesterol metabolic transformation in L-02 human hepatic cells via the PPARγ-ABCA1/CYP7A1 pathway. Food Funct..

[103] Luo J., Yang H., Song B.-L. (2020). Mechanisms and regulation of cholesterol homeostasis. Nat. Rev. Mol. Cell Biol..

[104] Maxfield F.R., Tabas I. (2005). Role of cholesterol and lipid organization in disease. Nature.

[105] Brown M.S., Radhakrishnan A., Goldstein J.L. (2018). Retrospective on Cholesterol Homeostasis: The Central Role of Scap. Annu. Rev. Biochem..

[106] Steinberg G.R., Kemp B.E. (2009). AMPK in Health and Disease. Physiol. Rev..

[107] Hardie D.G., Carling D. (1997). The AMP-Activated Protein Kinase. Fuel Gauge of the Mammalian Cell?. Eur. J. Biochem..

[108] Shin S.-K., Ha T.-Y., McGregor R.A., Choi M.-S. (2011). Long-term curcumin administration protects against atherosclerosis via hepatic regulation of lipoprotein cholesterol metabolism. Mol. Nutr. Food Res..

[109] Scharinger B., Messner B., Türkcan A., Schuster D., Vuorinen A., Pitterl F., Heinz K., Arnhard K., Laufer G., Grimm M. (2016). Leoligin, the major lignan from Edelweiss, inhibits 3-hydroxy-3-methyl-glutaryl-CoA reductase and reduces cholesterol levels in ApoE−/− mice. J. Mol. Cell. Cardiol..

[110] Zhao L.-Y., Huang W., Yuan Q.-X., Cheng J., Huang Z.-C., Ouyang L.-J., Zeng F.-H. (2012). Hypolipidaemic effects and mechanisms of the main component of Opuntia dillenii Haw. polysaccharides in high-fat emulsion-induced hyperlipidaemic rats. Food Chem..

[111] Chung M.J., Sung N.-J., Park C.-S., Kweon D.-K., Mantovani A., Moon T.-W., Lee S.-J., Park K.-H. (2008). Antioxidative and hypocholesterolemic activities of water-soluble puerarin glycosides in HepG2 cells and in C57 BL/6J mice. Eur. J. Pharmacol..

[112] Galle M., Kladniew B.R., Castro M.A., Villegas S.M., Lacunza E., Polo M., De Bravo M.G., Crespo R. (2015). Modulation by geraniol of gene expression involved in lipid metabolism leading to a reduction of serum-cholesterol and triglyceride levels. Phytomedicine.

[113] Ma S., Sun W., Gao L., Liu S. (2019). Therapeutic targets of hypercholesterolemia: HMGCR and LDLR. Diabetes Metab. Syndr. Obes. Targets Ther..

[114] Kim Y.-S., Lee Y.-M., Oh T.-I., Shin D.H., Kim G.-H., Kan S.-Y., Kang H., Kim J.H., Kim B.M., Yim W.J. (2018). Emodin Sensitizes Hepatocellular Carcinoma Cells to the Anti-Cancer Effect of Sorafenib through Suppression of Cholesterol Metabolism. Int. J. Mol. Sci..

[115] Grand-Perret T., Bouillot A., Perrot A., Commans S., Walker M., Issandou M. (2001). SCAP ligands are potent new lipid-lowering drugs. Nat. Med..

[116] Zhang Y., Ren P., Kang Q., Liu W., Li S., Li P., Liu H., Shang J., Zhang L., Gong Y. (2017). Effect of Tetramethylpyrazine on Atherosclerosis and SCAP/SREBP-1c Signaling Pathway in ApoE−/−Mice Fed with a High-Fat Diet. Evid.-Based Complement. Altern. Med..

[117] Cheng H., Xu N., Zhao W., Su J., Liang M., Xie Z., Wu X., Li Q. (2017). (−)-Epicatechin regulates blood lipids and attenuates hepatic steatosis in rats fed high-fat diet. Mol. Nutr. Food Res..

[118] Hajjaj H., Macé C., Roberts M., Niederberger P., Fay L.B. (2005). Effect of 26-Oxygenosterols from Ganoderma lucidum and Their Activity as Cholesterol Synthesis Inhibitors. Appl. Environ. Microbiol..

[119] Davidson M.H. (2007). Squalene synthase inhibition: A novel target for the management of dyslipidemia. Curr. Atheroscler. Rep..

[120] Chen Y., Chen X., Luo G., Zhang X., Lu F., Qiao L., He W., Li G., Zhang Y. (2018). Discovery of Potential Inhibitors of Squalene Synthase from Traditional Chinese Medicine Based on Virtual Screening and In Vitro Evaluation of Lipid-Lowering Effect. Molecules.

[121] Abubakar I., Tillmann T., Banerjee A. (2015). Global, regional, and national age-sex specific all-cause and cause-specific mortality for 240 causes of death, 1990–2013: A systematic analysis for the Global Burden of Disease Study 2013. Lancet.

[122] Bøhn S.K., Myhrstad M.C., Thoresen M., Holden M., Karlsen A., Tunheim S.H., Erlund I., Svendsen M., Seljeflot I., Moskaug J. (2010). Ø; et al. Blood cell gene expression associated with cellular stress defense is modulated by antioxidant-rich food in a randomised controlled clinical trial of male smokers. BMC Med..

[123] Anderson J.W., Baird P., Davis R.H., Ferreri S., Knudtson M., Koraym A., Waters V., Williams C.L. (2009). Health benefits of dietary fiber. Nutr. Rev..

[124] Islam S.U., Ahmed M.B., Ahsan H., Islam M., Shehzad A., Sonn J.K., Lee Y.S. (2020). An Update on the Role of Dietary Phytochemicals in Human Skin Cancer: New Insights into Molecular Mechanisms. Antioxidants.

[125] Alissa E.M., Ferns G.A. (2017). Dietary Fruits and Vegetables and Cardiovascular Diseases Risk. Crit. Rev. Food Sci. Nutr..

[126] Aune D., Giovannucci E., Boffetta P., Fadnes L.T., Keum N., Norat T., Greenwood D.C., Riboli E., Vatten L.J., Tonstad S. (2017). Fruit and vegetable intake and the risk of cardiovascular disease, total cancer and all-cause mortality—A systematic review and dose-response meta-analysis of prospective studies. Int. J. Epidemiol..

[127] Carmona-Jiménez Y., Palma M., Guillén-Sánchez D.A., García-Moreno M.V. (2021). Study of the Cluster Thinning Grape as a Source of Phenolic Compounds and Evaluation of Its Antioxidant Potential. Biomolecules.

[128] Cardoso L.M., Viana Leite J.P., Gouveia Peluzio M.D.C. (2011). Biological effects of anthocyanins on the atherosclerotic process. Rev. Colomb. Cienc. Químico-Farm..

[129] Castilla P., Echarri R., Dávalos A., Cerrato F., Ortega H., Teruel J.L., Lucas M.F., Gómez-Coronado D., Ortuño J., Lasunción M.A. (2006). Concentrated red grape juice exerts antioxidant, hypolipidemic, and antiinflammatory effects in both hemodialysis patients and healthy subjects. Am. J. Clin. Nutr..

[130] Vaisman N., Niv E. (2015). Daily consumption of red grape cell powder in a dietary dose improves cardiovascular parameters: A double blind, placebo-controlled, randomized study. Int. J. Food Sci. Nutr..

[131] Martins Â.M., Silva Sarto D.A.Q., Caproni K.D.P., Silva J., Silva J., Souza P.S., Dos Santos L., Ureña M.J.E., Souza Carvalho M.D.G.D., Vilas Boas B.M. (2020). Grape juice attenuates left ventricular hypertrophy in dyslipidemic mice. PLoS ONE.

[132] Van Mierlo L.A., Zock P.L., van der Knaap H.C., Draijer R. (2010). Grape polyphenols do not affect vascular function in healthy men. J. Nutr..

[133] Yubero N., Sanz-Buenhombre M., Guadarrama A., Villanueva S., Carrión J.M., Larrarte E., Moro C. (2013). LDL cholesterol-lowering effects of grape extract used as a dietary supplement on healthy volunteers. Int. J. Food Sci. Nutr..

[134] Zern T.L., Wood R.J., Greene C., West K.L., Liu Y., Aggarwal D., Shachter N.S., Fernandez M.L. (2005). Grape Polyphenols Exert a Cardioprotective Effect in Pre- and Postmenopausal Women by Lowering Plasma Lipids and Reducing Oxidative Stress. J. Nutr..

[135] Zunino S.J., Peerson J.M., Freytag T.L., Breksa A.P., Bonnel E.L., Woodhouse L.R., Storms D.H. (2014). Dietary grape powder increases IL-1β and IL-6 production by lipopolysaccharide-activated monocytes and reduces plasma concentrations of large LDL and large LDL-cholesterol particles in obese humans. Br. J. Nutr..

[136] Chiva-Blanch G., Urpi-Sarda M., Ros E., Arranz S., Valderas-Martínez P., Casas R., Sacanella E., Llorach R., Lamuela-Raventos R.M., Andres-Lacueva C. (2012). Dealcoholized Red Wine Decreases Systolic and Diastolic Blood Pressure and Increases Plasma Nitric Oxide. Circ. Res..

[137] Pourmasoumi M., Hadi A., Najafgholizadeh A., Joukar F., Mansour-Ghanaei F. (2020). The effects of cranberry on cardiovascular metabolic risk factors: A systematic review and meta-analysis. Clin. Nutr..

[138] Wilson T., Porcari J.P., Harbin D. (1998). Cranberry extract inhibits low density lipoprotein oxidation. Life Sci..

[139] Yu L.L., Zhou K.K., Parry J. (2005). Antioxidant properties of cold-pressed black caraway, carrot, cranberry, and hemp seed oils. Food Chem..

[140] Chu Y.-F., Liu R.H. (2005). Cranberries inhibit LDL oxidation and induce LDL receptor expression in hepatocytes. Life Sci..

[141] Reed J. (2002). Cranberry Flavonoids, Atherosclerosis and Cardiovascular Health. Crit. Rev. Food Sci. Nutr..

[142] Aviram M., Rosenblat M. (2013). Pomegranate for Your Cardiovascular Health. Rambam Maimonides Med. J..

[143] Fuhrman B., Volkova N., Aviram M. (2005). Pomegranate juice inhibits oxidized LDL uptake and cholesterol biosynthesis in macrophages. J. Nutr. Biochem..

[144] Boroushaki M.T., Mollazadeh H., Afshari A.R. (2016). Pomegranate seed oil: A comprehensive review on its therapeutic effects. Int. J. Pharm. Sci. Res..

[145] Esmaillzadeh A., Tahbaz F., Gaieni I., Alavi-Majd H., Azadbakht L. (2006). Cholesterol-Lowering Effect of Concentrated Pomegranate Juice Consumption in Type II Diabetic Patients with Hyperlipidemia. Int. J. Vitam. Nutr. Res..

[146] Aviram M., Dornfeld L., Rosenblat M., Volkova N., Kaplan M., Coleman R., Hayek T., Presser D., Fuhrman B. (2000). Pomegranate juice consumption reduces oxidative stress, atherogenic modifications to LDL, and platelet aggregation: Studies in humans and in atherosclerotic apolipoprotein E–deficient mice. Am. J. Clin. Nutr..

[147] Al-Moraie M.M., Arafat R.A., Al-Rasheedi A.A. (2013). Effect of pomegranate juice on lipid profile and antioxidant enzymes in hypercholesterolemic rats. Life Sci. J..

[148] Atrahimovich D., Khatib S., Sela S., Vaya J., Samson A.O. (2016). Punicalagin Induces Serum Low-Density Lipoprotein Influx to Macrophages. Oxidative Med. Cell. Longev..

[149] Tenore G.C., Caruso D., Buonomo G., D’Urso E., D’Avino M., Campiglia P., Marinelli L., Novellino E. (2017). Annurca (Malus pumilaMiller cv. Annurca) apple as a functional food for the contribution to a healthy balance of plasma cholesterol levels: Results of a randomized clinical trial. J. Sci. Food Agric..

[150] Nagasako-Akazome Y., Kanda T., Ohtake Y., Shimasaki H., Kobayashi T. (2007). Apple Polyphenols Influence Cholesterol Metabolism in Healthy Subjects with Relatively High Body Mass Index. J. Oleo Sci..

[151] Hyson D., Studebaker-Hallman D., Davis P.A., Gershwin M.E. (2000). Apple Juice Consumption Reduces Plasma Low-Density Lipoprotein Oxidation in Healthy Men and Women. J. Med. Food.

[152] Auclair S., Chironi G., Milenkovic D., Hollman P., Renard C., Mégnien J., Gariepy J., Paul J.-L., Simon A., Scalbert A. (2010). The regular consumption of a polyphenol-rich apple does not influence endothelial function: A randomised double-blind trial in hypercholesterolemic adults. Eur. J. Clin. Nutr..

[153] Vafa M.R., Haghighatjoo E., Shidfar F., Afshari S., Gohari M.R., Ziaee A. (2011). Effects of Apple Consumption on Lipid Profile of Hyperlipidemic and Overweight Men. Int. J. Prev. Med..

[154] Ravn-Haren G., Dragsted L.O., Buch-Andersen T., Jensen E.N., Jensen R.I., Németh-Balogh M., Paulovicsová B., Bergström A., Wilcks A., Licht T.R. (2013). Intake of whole apples or clear apple juice has contrasting effects on plasma lipids in healthy volunteers. Eur. J. Nutr..

[155] Barth S.W., Koch T.C.L., Watzl B., Dietrich H., Will F., Bub A. (2011). Moderate effects of apple juice consumption on obesity-related markers in obese men: Impact of diet–gene interaction on body fat content. Eur. J. Nutr..

[156] Kim Y., Keogh J.B., Clifton P.M. (2017). Benefits of Nut Consumption on Insulin Resistance and Cardiovascular Risk Factors: Multiple Potential Mechanisms of Actions. Nutrients.

[157] Bechthold A., Boeing H., Schwedhelm C., Hoffmann G., Knüppel S., Iqbal K., De Henauw S., Michels N., Devleesschauwer B., Schlesinger S. (2019). Food groups and risk of coronary heart disease, stroke and heart failure: A systematic review and dose-response meta-analysis of prospective studies. Crit. Rev. Food Sci. Nutr..

[158] Afshin A., Micha R., Khatibzadeh S., Mozaffarian D. (2014). Consumption of nuts and legumes and risk of incident ischemic heart disease, stroke, and diabetes: A systematic review and meta-analysis. Am. J. Clin. Nutr..

[159] Zhou D., Yu H., He F., Reilly K.H., Zhang J., Li S., Zhang T., Wang B., Ding Y., Xi B. (2014). Nut consumption in relation to cardiovascular disease risk and type 2 diabetes: A systematic review and meta-analysis of prospective studies. Am. J. Clin. Nutr..

[160] Freeman A.M., Morris P.B., Barnard N., Esselstyn C.B., Ros E., Agatston A., Devries S., O’Keefe J., Miller M., Ornish D. (2017). Trending Cardiovascular Nutrition Controversies. J. Am. Coll. Cardiol..

[161] Holmes M.V., Asselbergs F.W., Palmer T.M., Drenos F., Lanktree M.B., Nelson C.P., Dale C.E., Padmanabhan S., Finan C., Swerdlow D.I. (2015). Mendelian randomization of blood lipids for coronary heart disease. Eur. Heart J..

[162] Griel A.E., Kris-Etherton P.M. (2006). Tree nuts and the lipid profile: A review of clinical studies. Br. J. Nutr..

[163] Zhao J.V., Schooling C.M. (2019). Effect of linoleic acid on ischemic heart disease and its risk factors: A Mendelian randomization study. BMC Med..

[164] Trautwein E.A., McKay S. (2020). The Role of Specific Components of a Plant-Based Diet in Management of Dyslipidemia and the Impact on Cardiovascular Risk. Nutrients.

[165] Xu X.-R., Zou Z.-Y., Xiao X., Huang Y.-M., Wang X., Lin X.-M. (2013). Effects of Lutein Supplement on Serum Inflammatory Cytokines, ApoE and Lipid Profiles in Early Atherosclerosis Population. J. Atheroscler. Thromb..

[166] García-Conesa M.T., Chambers K., Combet E., Pinto P., Garcia-Aloy M., Andres-Lacueva C., De Pascual-Teresa S., Mena P., Ristic A.K., Hollands W.J. (2018). Meta-Analysis of the Effects of Foods and Derived Products Containing Ellagitannins and Anthocyanins on Cardiometabolic Biomarkers: Analysis of Factors Influencing Variability of the Individual Responses. Int. J. Mol. Sci..

[167] Hernández-Alonso P., Giardina S., Salas-Salvadó J., Arcelin P., Bulló M. (2016). Chronic pistachio intake modulates circulating microRNAs related to glucose metabolism and insulin resistance in prediabetic subjects. Eur. J. Nutr..

[168] Ortega F.J., Cardona-Alvarado M.I., Mercader J.M., Moreno-Navarrete J.M., Moreno M., Sabater M., Fuentes-Batllevell N., Ramírez-Chávez E., Ricart W., Molina-Torres J. (2015). Circulating profiling reveals the effect of a polyunsaturated fatty acid-enriched diet on common microRNAs. J. Nutr. Biochem..

[169] Del Gobbo L.C., Falk M.C., Feldman R., Lewis K., Mozaffarian D. (2015). Effects of tree nuts on blood lipids, apolipoproteins, and blood pressure: Systematic review, meta-analysis, and dose-response of 61 controlled intervention trials. Am. J. Clin. Nutr..

[170] Guasch-Ferré M., Li J., Hu F.B., Salas-Salvadó J., Tobias D.K. (2018). Effects of walnut consumption on blood lipids and other cardiovascular risk factors: An updated meta-analysis and systematic review of controlled trials. Am. J. Clin. Nutr..

[171] Musa-Veloso K., Paulionis L., Poon T., Lee H.Y. (2016). The effects of almond consumption on fasting blood lipid levels: A systematic review and meta-analysis of randomised controlled trials. J. Nutr. Sci..

[172] Njike V.Y., Ayettey R., Petraro P., Treu J.A., Katz D.L. (2015). Walnut ingestion in adults at risk for diabetes: Effects on body composition, diet quality, and cardiac risk measures. BMJ Open Diabetes Res. Care.

[173] Flores-Mateo G., Rojas-Rueda D., Basora J., Ros E., Salas-Salvadó J. (2013). Nut intake and adiposity: Meta-analysis of clinical trials. Am. J. Clin. Nutr..

[174] Schwingshackl L., Schwarzer G., Rücker G., Meerpohl J.J. (2019). Perspective: Network Meta-analysis Reaches Nutrition Research: Current Status, Scientific Concepts, and Future Directions. Adv. Nutr..

[175] Hutton B., Salanti G., Caldwell D.M., Chaimani A., Schmid C.H., Cameron C., Ioannidis J.P., Straus S.E., Thorlund K., Jansen J.P. (2015). The PRISMA Extension Statement for Reporting of Systematic Reviews Incorporating Network Meta-analyses of Health Care Interventions: Checklist and Explanations. Ann. Intern. Med..

[176] Liu K., Hui S., Wang B., Kaliannan K., Guo X., Liang L. (2020). Comparative effects of different types of tree nut consumption on blood lipids: A network meta-analysis of clinical trials. Am. J. Clin. Nutr..

[177] Gouws C., Mortazavi R., Mellor D., McKune A., Naumovski N. (2020). The effects of Prickly Pear fruit and cladode (*Opuntia* spp.) consumption on blood lipids: A systematic review. Complement. Ther. Med..

[178] Palumbo B., Efthimiou Y., Stamatopoulos J., Oguogho A., Budinsky A., Palumbo R., Sinzinger H. (2003). Prickly pear induces upregulation of liver LDL binding in familial heterozygous hypercholesterolemia. Nucl. Med. Rev..

[179] Khouloud A., Abedelmalek S., Chtourou H., Souissi N. (2017). The effect of Opuntia ficus-indica juice supplementation on oxidative stress, cardiovascular parameters, and biochemical markers following yo-yo Intermittent recovery test. Food Sci. Nutr..

[180] Wolfram R.M., Kritz H., Efthimiou Y., Stomatopoulos J., Sinzinger H. (2002). Effect of prickly pear (Opuntia robusta) on glucose- and lipid-metabolism in non-diabetics with hyperlipidemia--a pilot study. Wien. Klin. Wochenschr..

[181] Goh K.K.T., Pinder D.N., Hall C.E., Hemar Y. (2006). Rheological and Light Scattering Properties of Flaxseed Polysaccharide Aqueous Solutions. Biomacromolecules.

[182] Kristensen M., Jensen M.G., Aarestrup J., Petersen K.E., Søndergaard L., Mikkelsen M.S., Astrup A. (2012). Flaxseed dietary fibers lower cholesterol and increase fecal fat excretion, but magnitude of effect depend on food type. Nutr. Metab..

[183] Surampudi P., Enkhmaa B., Anuurad E., Berglund L. (2016). Lipid Lowering with Soluble Dietary Fiber. Curr. Atheroscler. Rep..

[184] Zhang W., Wang X., Liu Y., Tian H., Flickinger B., Empie M.W., Sun S.Z. (2008). Dietary flaxseed lignan extract lowers plasma cholesterol and glucose concentrations in hypercholesterolaemic subjects. Br. J. Nutr..

[185] Arjmandi B.H., Khan D.A., Juma S., Drum M.L., Venkatesh S., Sohn E., Wei L., Derman R. (1998). Whole flaxseed consumption lowers serum LDL-cholesterol and lipoprotein(a) concentrations in postmenopausal women. Nutr. Res..

[186] Edel A.L., Rodriguez-Leyva D., Maddaford T.G., Caligiuri S.P., Austria J.A., Weighell W., Guzman R., Aliani M., Pierce G.N. (2015). Dietary Flaxseed Independently Lowers Circulating Cholesterol and Lowers It beyond the Effects of Cholesterol-Lowering Medications Alone in Patients with Peripheral Artery Disease. J. Nutr..

[187] Vaidean G.D., Manczuk M., Vansal S.S., Griffith J. (2018). The cholesterol-lowering effect of statins is potentiated by whole grains intake. The Polish Norwegian Study (PONS). Eur. J. Intern. Med..

[188] Temple N.J. (2018). Fat, Sugar, Whole Grains and Heart Disease: 50 Years of Confusion. Nutrients.

[189] Hollænder P.L., Ross A.B., Kristensen M. (2015). Whole-grain and blood lipid changes in apparently healthy adults: A systematic review and meta-analysis of randomized controlled studies. Am. J. Clin. Nutr..

[190] Fatahi S., Daneshzad E., Kord-Varkaneh H., Bellissimo N., Brett N.R., Azadbakht L. (2018). Impact of Diets Rich in Whole Grains and Fruits and Vegetables on Cardiovascular Risk Factors in Overweight and Obese Women: A Randomized Clinical Feeding Trial. J. Am. Coll. Nutr..

[191] Helnæs A., Kyrø C., Andersen I., Lacoppidan S., Overvad K., Christensen J., Tjønneland A., Olsen A. (2016). Intake of whole grains is associated with lower risk of myocardial infarction: The Danish Diet, Cancer and Health Cohort. Am. J. Clin. Nutr..

[192] Blanco Mejia S., Messina M., Li S.S., Viguiliouk E., Chiavaroli L., Khan T.A., Srichaikul K., Mirrahimi A., Sievenpiper J.L., Kris-Etherton P. (2019). A meta-analysis of 46 studies identified by the FDA demonstrates that soy protein decreases circulating LDL and total cholesterol concentrations in adults. J. Nutr..

[193] Høie L.H., Morgenstern E.C., Grünwald J., Graubaum H.J., Busch R., Lüder W., Zunft H.J. (2005). A double-blind placebo-controlled clinical trial compares the cholesterollowering effects of two different soy protein preparations in hypercholesterolemic subjects. Eur. J. Nutr..

[194] Lukaczer D., DeAnn J.L., Lerman R.H., Darland G., Schiltz B., Tripp M., Bland J.S. (2006). Effect of a low glycemic index diet with soy protein and phytosterols on CVD risk factors in postmenopausal women. Nutrition.

[195] Taku K., Umegaki K., Sato Y., Taki Y., Endoh K., Watanabe S. (2007). Soy isoflavones lower serum total and LDL cholesterol in humans: A meta-analysis of 11 randomized controlled trials. Am. J. Clin. Nutr..

[196] Zhan S., Ho S.C. (2005). Meta-analysis of the effects of soy protein containing isoflavones on the lipid profile. Am. J. Clin. Nutr..

[197] Tokede O.A., Onabanjo T.A., Yansane A., Gaziano J.M., Djoussé L. (2015). Soya products and serum lipids: A meta-analysis of randomised controlled trials. Br. J. Nutr..

[198] Kim J., Lee H., Lee O., Lee K.-H., Lee Y.-B., Young K.D., Jeong Y.H., Choue R. (2013). Isoflavone supplementation influenced levels of triglyceride and luteunizing hormone in Korean postmenopausal women. Arch. Pharmacal Res..

[199] Qin Y., Shu F., Zeng Y., Meng X., Wang B., Diao L., Wang L., Wan J., Zhu J., Wang J. (2014). Daidzein Supplementation Decreases Serum Triglyceride and Uric Acid Concentrations in Hypercholesterolemic Adults with the Effect on Triglycerides Being Greater in Those with the GA Compared with the GG Genotype of ESR-β Rsa I. J. Nutr..

[200] Usui T., Tochiya M., Sasaki Y., Muranaka K., Yamakage H., Himeno A., Shimatsu A., Inaguma A., Ueno T., Uchiyama S. (2013). Effects of naturalS-equol supplements on overweight or obesity and metabolic syndrome in the Japanese, based on sex and equol status. Clin. Endocrinol..

[201] Squadrito F., Marini H., Bitto A., Altavilla D., Polito F., Adamo E.B., D’Anna R., Arcoraci V., Burnett B.P., Minutoli L. (2013). Genistein in the Metabolic Syndrome: Results of a Randomized Clinical Trial. J. Clin. Endocrinol. Metab..

[202] Key T.J., Appleby P.N., Rosell M.S. (2006). Health effects of vegetarian and vegan diets. Proc. Nutr. Soc..

[203] Ashen M.D. (2013). Vegetarian diets in cardiovascular prevention. Curr. Treat. Options Cardiovasc. Med..

[204] Anderson T.J., Grégoire J., Pearson G.J., Barry A.R., Couture P., Dawes M., Francis G.A., Genest J., Grover S., Gupta M. (2016). 2016 Canadian Cardiovascular Society Guidelines for the Management of Dyslipidemia for the Prevention of Cardiovascular Disease in the Adult. Can. J. Cardiol..

[205] Sterling S.R., Bowen S.-A. (2019). The potential for plant-based diets to promote health among blacks living in the United States. Nutrients.

[206] Larsson S.C., Orsini N. (2014). Red Meat and Processed Meat Consumption and All-Cause Mortality: A Meta-Analysis. Am. J. Epidemiol..

[207] Kwok C.S., Umar S., Myint P.K., Mamas M.A., Loke Y.K. (2014). Vegetarian diet, Seventh Day Adventists and risk of cardiovascular mortality: A systematic review and meta-analysis. Int. J. Cardiol..

[208] Migliaccio S., Brasacchio C., Pivari F., Salzano C., Barrea L., Muscogiuri G., Savastano S., Colao A. (2020). What is the best diet for cardiovascular wellness? A comparison of different nutritional models. Int. J. Obes. Suppl..

[209] Crowe F.L., Appleby P.N., Travis R.C., Key T.J. (2013). Risk of hospitalization or death from ischemic heart disease among British vegetarians and nonvegetarians: Results from the EPIC-Oxford cohort study. Am. J. Clin. Nutr..

[210] Liu R.H. (2013). Dietary Bioactive Compounds and Their Health Implications. J. Food Sci..

[211] Wang F., Zheng J., Yang B., Jiang J., Fu Y., Li D. (2015). Effects of Vegetarian Diets on Blood Lipids: A Systematic Review and Meta-Analysis of Randomized Controlled Trials. J. Am. Heart Assoc..

[212] Key T.J., Fraser G.E., Thorogood M., Appleby P.N., Beral V., Reeves G., Burr M.L., Chang-Claude J., Frentzel-Beyme R., Kuzma J.W. (1999). Mortality in vegetarians and nonvegetarians: Detailed findings from a collaborative analysis of 5 prospective studies. Am. J. Clin. Nutr..

[213] Chen C., Lin Y., Lin T., Lin C., Chen B., Lin C. (2008). Total cardiovascular risk profile of Taiwanese vegetarians. Eur. J. Clin. Nutr..

[214] Jones J.R., Lineback D.M., Levine M.J. (2006). Dietary reference intakes: Implications for fiber labeling and consumption: A summary of the International Life Sciences Institute North America Fiber Workshop, June 1–2, 2004, Washington, DC. Nutr. Rev..

[215] Lia A., Hallmans G., Sandberg A.S., Sundberg B., Aman P., Andersson H. (1995). Oat beta-glucan increases bile acid excretion and a fiber-rich barley fraction increases cholesterol excretion in ileostomy subjects. Am. J. Clin. Nutr..

[216] Brown L., Rosner B., Willett W.W., Sacks F.M. (1999). Cholesterol-lowering effects of dietary fiber: A meta-analysis. Am. J. Clin. Nutr..

[217] Evans C.E., Greenwood D.C., Threapleton D.E., Cleghorn C.L., Nykjaer C., Woodhead C.E., Gale C.P., Burley V.J. (2015). Effects of dietary fibre type on blood pressure: A systematic review and meta-analysis of randomized controlled trials of healthy individuals. J. Hypertens..

[218] Kim Y., Je Y. (2016). Dietary fibre intake and mortality from cardiovascular disease and all cancers: A meta-analysis of prospective cohort studies. Arch. Cardiovasc. Dis..

[219] McKeown N.M., Meigs J.B., Liu S., Saltzman E., Wilson P.W., Jacques P.F. (2004). Carbohydrate Nutrition, Insulin Resistance, and the Prevalence of the Metabolic Syndrome in the Framingham Offspring Cohort. Diabetes Care.

[220] Bazzano L.A., Thompson A.M., Tees M.T., Nguyen C.H., Winham D.M. (2011). Non-soy legume consumption lowers cholesterol levels: A meta-analysis of randomized controlled trials. Nutr. Metab. Cardiovasc. Dis..

[221] Marques F.Z., Nelson E., Chu P.-Y., Horlock D., Fiedler A., Ziemann M., Tan J.K., Kuruppu S., Rajapakse N.W., El-Osta A. (2017). High-Fiber Diet and Acetate Supplementation Change the Gut Microbiota and Prevent the Development of Hypertension and Heart Failure in Hypertensive Mice. Circulation.

[222] Whitehead A., Beck E.J., Tosh S., Wolever T.M. (2014). Cholesterol-lowering effects of oat β-glucan: A meta-analysis of randomized controlled trials. Am. J. Clin. Nutr..

[223] Mirmiran P., Bahadoran Z., Khalili Moghadam S., Zadeh Vakili A., Azizi F. (2016). A Prospective Study of Different Types of Dietary Fiber and Risk of Cardiovascular Disease: Tehran Lipid and Glucose Study. Nutrients.

[224] Threapleton D.E., Greenwood D.C., Evans C.E., Cleghorn C.L., Nykjaer C., Woodhead C., Cade J.E., Gale C.P., Burley V.J. (2013). Dietary fibre intake and risk of cardiovascular disease: Systematic review and meta-analysis. BMJ.

[225] Greenwood D.C., Threapleton D.E., Evans C.E., Cleghorn C.L., Nykjaer C., Woodhead C., Burley V.J. (2013). Glycemic Index, Glycemic Load, Carbohydrates, and Type 2 Diabetes: Systematic review and dose-response meta-analysis of prospective studies. Diabetes Care.

[226] Khan K., Jovanovski E., Ho H.V.T., Marques A.C.R., Zurbau A., Mejia S.B., Sievenpiper J.L., Vuksan V. (2018). The effect of viscous soluble fiber on blood pressure: A systematic review and meta-analysis of randomized controlled trials. Nutr. Metab. Cardiovasc. Dis..

[227] Evans C.E.L. (2020). Dietary fibre and cardiovascular health: A review of current evidence and policy. Proc. Nutr. Soc..

[228] Den Besten G., Van Eunen K., Groen A.K., Venema K., Reijngoud D.-J., Bakker B.M. (2013). The role of short-chain fatty acids in the interplay between diet, gut microbiota, and host energy metabolism. J. Lipid Res..

[229] Ban S.J., Rico C.W., Um I.C., Kang M.Y. (2012). Comparative evaluation of the hypolipidemic effects of hydroxyethyl methylcellulose (HEMC) and hydroxypropyl methylcellulose (HPMC) in high fat-fed mice. Food Chem. Toxicol..

[230] Liu X., Yang F., Song T., Zeng A., Wang Q., Sun Z., Shen J. (2012). Therapeutic Effect of Carboxymethylated and Quanternized Chitosan on Insulin Resistance in High-Fat-Diet-Induced Rats and 3T3-L1 Adipocytes. J. Biomater. Sci. Polym. Ed..

[231] (1984). Lipid Research Clinics Coronary Primary Prevention Trial Results. II. The relationship of reduction in incidence of coronary heart disease to cholesterol lowering. JAMA.

[232] Mathews R., Kamil A., Chu Y. (2020). Global review of heart health claims for oat beta-glucan products. Nutr. Rev..

[233] McRorie J., Fahey G., Wallace T. (2015). Fiber supplements and clinically meaningful health benefits: Identifying the physiochemical characteristics of fiber that drive specific physiologic effects. The CRC Handbook on Dietary Supplements in Health Promotion.

[234] Wolever T.M., Tosh S.M., Gibbs A.L., Brand-Miller J., Duncan A.M., Hart V., Lamarche B., Thomson B.A., Duss R., Wood P.J. (2010). Physicochemical properties of oat β-glucan influence its ability to reduce serum LDL cholesterol in humans: A randomized clinical trial. Am. J. Clin. Nutr..

[235] Comerford K.B., Artiss J.D., Jen K.-L.C., Karakas S.E. (2011). The Beneficial Effects α-Cyclodextrin on Blood Lipids and Weight Loss in Healthy Humans. Obesity.

[236] Jarosz P.A., Fletcher E., Elserafy E., Artiss J.D., Jen K.-L.C. (2013). The Effect of α-Cyclodextrin on postprandial lipid and glycemic responses to a fat-containing meal. Metabolism.

[237] Hemler E.C., Hu F.B. (2019). Plant-Based Diets for Cardiovascular Disease Prevention: All Plant Foods Are Not Created Equal. Curr. Atheroscler. Rep..

[238] Zhou D.-D., Luo M., Shang A., Mao Q.-Q., Li B.-Y., Gan R.-Y., Li H.-B. (2021). Antioxidant Food Components for the Prevention and Treatment of Cardiovascular Diseases: Effects, Mechanisms, and Clinical Studies. Oxidative Med. Cell. Longev..

[239] Zock P.L., Blom W.A., Nettleton J.A., Hornstra G. (2016). Progressing Insights into the Role of Dietary Fats in the Prevention of Cardiovascular Disease. Curr. Cardiol. Rep..

[240] Schwingshackl L., Bogensberger B., Benčič A., Knüppel S., Boeing H., Hoffmann G. (2018). Effects of oils and solid fats on blood lipids: A systematic review and network meta-analysis. J. Lipid Res..

[241] Theuwissen E., Mensink R.P. (2008). Water-soluble dietary fibers and cardiovascular disease. Physiol. Behav..

[242] Gylling H., Plat J., Turley S.D., Ginsberg H.N., Ellegård L., Jessup W., Jones P.J.H., Lütjohann D., März W., Masana L. (2014). Plant sterols and plant stanols in the management of dyslipidaemia and prevention of cardiovascular disease. Atherosclerosis.

[243] Satija A., Bhupathiraju S.N., Spiegelman D., Chiuve S.E., Manson J.E., Willett W., Rexrode K.M., Rimm E.B., Hu F.B. (2017). Healthful and Unhealthful Plant-Based Diets and the Risk of Coronary Heart Disease in U.S. Adults. J. Am. Coll. Cardiol..

[244] Willett W., Rockström J., Loken B., Springmann M., Lang T., Vermeulen S., Garnett T., Tilman D., DeClerck F., Wood A. (2019). Food in the Anthropocene: The EAT–Lancet Commission on healthy diets from sustainable food systems. Lancet.

[245] Corrin T., Papadopoulos A. (2017). Understanding the attitudes and perceptions of vegetarian and plant-based diets to shape future health promotion programs. Appetite.

[246] Fehér A., Gazdecki M., Véha M., Szakály M., Szakály Z. (2020). A Comprehensive Review of the Benefits of and the Barriers to the Switch to a Plant-Based Diet. Sustainability.

